# Energy Landscape Sampling Reveals Ligand-Dependent Structural Dynamics of the Protease-Activated Receptor 1

**DOI:** 10.1021/acs.jcim.6c01315

**Published:** 2026-09-23

**Authors:** Inken Kaja Schwerin, Shihai Jiang, Claudia Stäubert, Berend Isermann, Georg Künze

**Affiliations:** † Institute for Drug Discovery, 9180 Leipzig University, Brüderstraße 34, 04103 Leipzig, Germany; ‡ Institute of Laboratory Medicine, Clinical Chemistry and Molecular Diagnostic, 39066 University Hospital Leipzig, Paul-List-Straße 13-15, 04103 Leipzig, Germany; § Rudolf Schönheimer Institute of Biochemistry, Molecular Biochemistry, Medical Faculty, Leipzig University, Johannisallee 30, 04103 Leipzig, Germany; ∥ LeiCeM—Leipzig Center of Metabolism, Leipzig University, 04103 Leipzig, Germany; ⊥ Interdisciplinary Center for Bioinformatics, Leipzig University, 04107 Leipzig, Germany; # Center for Scalable Data Analytics and Artificial Intelligence, Leipzig University, 04105 Leipzig, Germany

## Abstract

Protease-activated receptor 1 (PAR1) is a class A G protein-coupled receptor activated by proteolytic cleavage and exposure of a tethered agonist. While existing structures have revealed inactive and active conformations, the ligand-dependent conformational energy landscape governing PAR1 activation remains poorly understood. Here, we employed enhanced-sampling molecular dynamics simulations using the string method with swarms of trajectories to quantify how extracellular ligands and G-protein binding modulate PAR1 activation. Free-energy landscapes were computed for five receptor states (apo, tethered agonist, thrombin receptor-activating peptide (TRAP), vorapaxar (VPX), and VPX + Na^+^) using five transmembrane (TM) helix distance metrics as collective variables. In the apo state, PAR1 samples inactive, intermediate, and active-like conformations, indicating substantial intrinsic receptor flexibility. VPX increases the free energy of active conformations and restricts TM helix 5 and 6 rearrangements, while VPX + Na^+^ eliminates intermediate basins and stabilizes the inactive state. In contrast, the tethered agonist stabilizes the active state as the global minimum and promotes TM6 displacement of up to ∼1 nm. The simulations reveal a continuous activation pathway linking extracellular ligand engagement to intracellular motifs, including the connector, DRF, intracellular loop 3 (ICL3), and KRK motifs, with intracellular chloride ion coordination contributing to inactive-state stabilization. Gα_q_ binding reduces TM6 flexibility and shifts the free-energy minimum toward more active-like conformations. Alanine mutagenesis and G_q_-CASE BRET assays validate predicted roles of key residues in activation and G-protein coupling. Together, these results provide a detailed understanding of ligand- and transducer-dependent PAR1 signaling and inform the design of pathway-selective PAR1 modulators.

## Graphical abstract

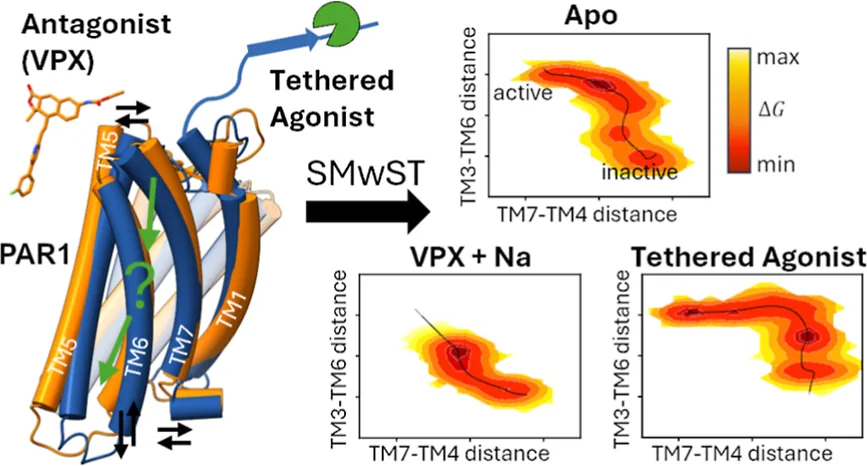

## Introduction

G protein-coupled receptors (GPCRs) constitute the largest family of membrane-embedded signaling proteins in the human genome and regulate a vast array of physiological processes, including neurotransmission, immune responses, cardiovascular function, and hemostasis.^1^ Their central role in transducing extracellular signals into intracellular biochemical responses has established GPCRs as major pharmacological targets, underscoring the importance of understanding GPCR structure, activation, and signaling mechanisms.^2^

Within this superfamily, the protease-activated receptors (PARs), including PAR1 through PAR4, play essential roles in vascular biology, inflammation, coagulation, and tissue repair.^3,4^ Among them, PAR1 is the prototypical and most extensively studied member. PAR1 is expressed on platelets, endothelial cells, and various other cell types, where it senses proteolytic activity in the extracellular environment and translates that into cellular responses (e.g., thrombosis, inflammation, endothelial barrier regulation).^5,6^

Unlike canonical GPCRs activated by diffusible ligands, PARs are activated through a unique proteolytic mechanism in which cleavage of the N-terminal extracellular domain generates a new N-terminus that acts as a “tethered agonist”, folding back into the orthosteric binding pocket to trigger receptor activation. This irreversible cleavage and self-activation mechanism distinguishes PARs from most GPCRs.^6,7^ In case of PAR1, canonical activation occurs via thrombin cleavage at Arg41, while noncanonical activation by the coagulation protease activated protein C (aPC) occurs at Arg46. The different tethered ligands generated can, in turn, initiate different intracellular signaling programs.^5,8^

This protease-dependent activation underlies a phenomenon known as biased signaling. Thrombin-activated PAR1 typically couples to heterotrimeric G proteins (such as G_q_, G_i_, or G_12/13_) to mediate platelet activation, pro-inflammatory responses, and cytoskeletal changes; in contrast, aPC-cleaved PAR1 preferentially engages β-arrestin-dependent or alternative signaling pathways leading to cytoprotection, anti-inflammatory output, and endothelial barrier stabilization. Because PAR1 is implicated in both harmful (thrombotic, inflammatory) and beneficial (cytoprotective) processes, the ability to selectively modulate PAR1 signaling outputs, e.g., by favoring protective over detrimental pathways, is of great pharmacological interest.^5,8,9^ In that context, allosteric modulators such as lipid-anchored intracellular peptides (so-called pepducins)^10,11^ and small molecules known as parmodulins have been developed to bias PAR1 signaling rather than to simply block canonical orthosteric activation.^12–14^

Structural studies have contributed key insights into how different ligands, both synthetic antagonists and natural tethered agonists, engage PAR1. For example, the crystal structure of human PAR1 bound to the antagonist vorapaxar (VPX) revealed an unusual mode of small-molecule binding. Rather than occupying a deep, solvent-exposed pocket typical for many GPCRs, VPX binds in a shallow, membrane-proximal binding pocket with minimal exposure to extracellular solvent, consistent with its essentially irreversible antagonism of PAR1.^15^ This VPX-bound structure represents an intermediate receptor conformation according to the GPCRdb^16^ activation-state classification (activation score 56%) and contains a sodium ion bound at a conserved site in the center of the transmembrane (TM) bundle (Figure 1).

**Figure 1:**
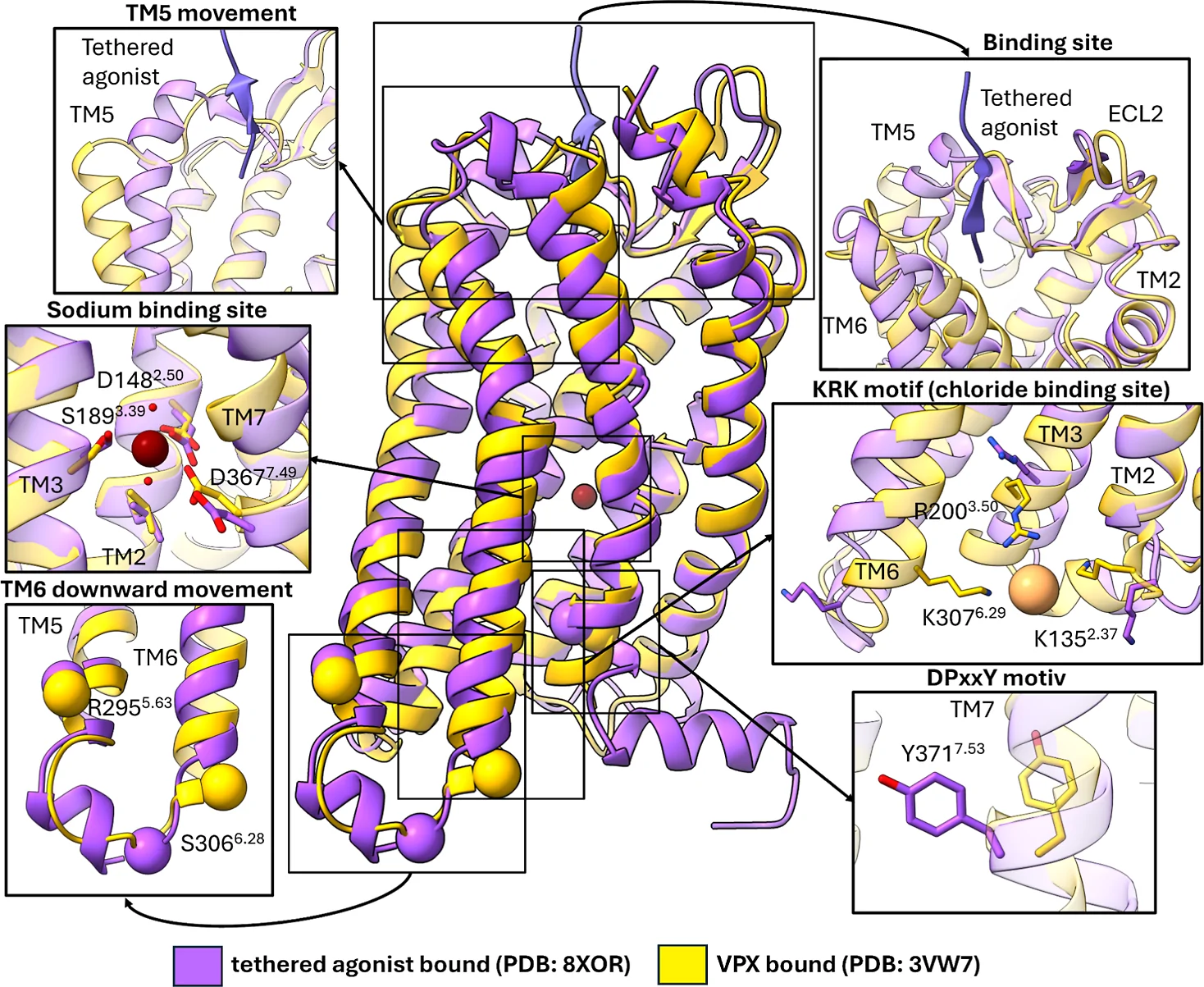
Comparison of tethered agonist- and VPX-bound PAR1 structures reveals key structural features associated with receptor activation. Engagement of the tethered thrombin agonist with the extracellular binding pocket via β-strand pairing with ECL2 (upper right) initiates a cascade of activation-associated conformational changes. These include inward movement of TM5 and compaction of the ligand-binding pocket (upper left), downward displacement of TM6 (lower left), and an inward twist of TM7 at the conserved NPxxY motif (DPxxY in PAR1) (lower right), collectively creating space for G-protein binding. Activation is further accompanied by displacement of a chloride ion from a conserved intracellular KRK motif (middle right) and of a sodium ion from a conserved site within the core of the TM bundle (middle left), which is occupied in the inactive VPX-bound structure.

More recently, high-resolution cryo-electron microscopy (cryo-EM) structures of activated PAR1 in complex with heterotrimeric G proteins revealed a strikingly unconventional conformational activation mechanism summarized in Figure 1.^17^ In these structures, binding of the thrombin-generated tethered agonist to a shallow extracellular pocket, mediated by β-strand pairing with extracellular loop 2 (ECL2) (Figure 1), triggers a coordinated series of conformational transitions. These include inward movement of TM helix 5 (TM5) and compaction of the extracellular binding pocket, a downward displacement of TM6, and an inward twist of TM7 at the conserved NPxxY motif (DPxxY in PAR1), which together create space for G-protein binding (Figure 1). Concomitantly, a chloride ion is displaced from a conserved intracellular KRK motif, and the sodium ion observed in the inactive VPX-bound structure is expelled from the core of the TM bundle (Figure 1). Notably, the β-sheet-like interaction between the tethered agonist and ECL2 represents a binding mode not observed in other tethered-agonist-activated receptors.^17^

Although these structural snapshots provide unprecedented insight into PAR1 activation, they leave open key mechanistic questions: how do different ligands (e.g., small-molecule antagonists, synthetic peptides, natural tethered agonists) modulate the conformational energy landscape of PAR1? Which microswitch motifs and intramolecular interactions are stabilized or destabilized? Importantly, how do these different ligands influence the dynamic equilibrium among inactive, intermediate, and active receptor states in the presence or absence of intracellular G proteins? In other words, what is the full repertoire of PAR1’s conformational ensembles under different ligand and signaling contexts?

Molecular dynamics (MD) simulations, especially enhanced-sampling MD approaches, provide a powerful complement to static structures by enabling the exploration of GPCR dynamics, conformational transitions, and ligand-dependent modulation of receptor ensembles over relevant time scales.^18,19^ Indeed, enhanced-sampling MD techniques (e.g., metadynamics, accelerated MD, swarm-of-trajectories, string methods)^20,21^ have been successfully applied to study activation dynamics in several GPCRs. For instance, agonist- and antagonist-bound states of the β_2_-adrenergic receptor (β_2_AR) were characterized using accelerated MD to reveal transitions between inactive and active conformations;^22^ and metadynamics-based protocols have been developed to determine ligand binding modes and free-energy profiles for the A2A adenosine receptor (A_2_AR).^23^ These studies demonstrate how ligand identity reshapes the conformational ensemble, affects microswitch networks, and can bias transducer coupling.

In the present study, we apply the string method with swarms of trajectories (SMwST)^24–26^ to simulate the activation dynamics of PAR1 under varying ligand and signaling conditions. Specifically, we probe the effects of three extracellular ligands: the antagonist VPX, a synthetic peptide agonist derived from the tethered ligand, thrombin receptor-activating peptide (TRAP), and the endogenous tethered agonist. In addition, we include the Gα_q_ subunit in the simulations to assess how receptor-transducer interactions shape the energy landscape. Our simulations broadly sample the conformational energy landscape of PAR1 and reveal how different ligands (and the presence vs absence of Gα_q_) stabilize or destabilize distinct residue motifs and microswitch networks critical for PAR1 activation.

Finally, to validate key predictions from our computational analysis, we performed biochemical characterization using site-directed mutagenesis combined with G-protein dissociation assays. These experiments confirm the relevance of specific residues and interactions highlighted by our simulations, providing an integrated mechanistic understanding of how ligand-dependent modulation of the PAR1 energy landscape controls receptor activation.

## Results and Discussion

### Ligand-Dependent Modulation of the PAR1 Activation Free-Energy Landscape

To enable a systematic comparison of PAR1 activation dynamics and the associated conformational free-energy landscapes across distinct ligand-bound states, we investigated five receptor conditions: PAR1 bound to the tethered agonist generated by thrombin cleavage, PAR1 bound to the TRAP peptide corresponding to the first six residues of the tethered agonist (SFLLRN), the ligand-free (apo) receptor, PAR1 bound to the antagonist VPX, and PAR1 bound to VPX in the presence of a sodium ion located within the TM helix bundle, a known negative allosteric modulator of class A GPCRs.^27^ MD simulation systems for all five conditions were prepared as described in the Methods section. Transitions between inactive and active receptor states were explored using the SMwST method, an enhanced sampling approach previously applied to the β_2_AR^26,28^ and other membrane proteins.^29^

Initial transition paths approximating the PAR1 activation pathway were generated using targeted MD (tMD) (see Methods), which was employed to sample conformations between three reference structures: the G_q_-bound active PAR1 cryo-EM structure,^17^ the VPX-bound intermediate-state PAR1 crystal structure,^15^ and an AlphaFold-generated PAR1 model obtained from GPCRdb. The selection of this model as the inactive end point was based on a comparative analysis of available experimental structures and activation-state descriptors (Figure S1), including key activation motifs and the a100 index, as described in Methods. The a100 index is defined as a linear combination of five interhelical Cα–Cα distances and has been shown to reliably quantify the activation state of class A GPCRs.^30^ In the present study, the a100 index was used as a collective variable (CV) to characterize receptor activation and deactivation along the free-energy landscape. In addition, five further CVs defined as interhelical Cα–Cα distances were employed as progress coordinates to guide SMwST sampling. This CV set was previously derived by Fleetwood et al. based on feature importance analysis of long unbiased (de)­activation simulations of the β_2_AR.^28^ The corresponding PAR1 residue pairs defining these CVs are listed in Table S1. We confirmed that the combined set of CVs reliably distinguishes inactive, intermediate, and active PAR1 conformations (Figure S2). For each receptor system, 300 iterations of string optimization were performed using 42 configurations distributed along the string, which was sufficient to achieve convergence (Figure S3). Trajectories from the final iterations were subsequently used to compute free-energy landscapes in the vicinity of the most probable activation pathway and to project these onto additional progress coordinates beyond the original CV set, enabling a more detailed analysis of PAR1 activation. Analysis of ligand rmsd values confirmed stable ligand binding in all ligand-bound systems, and no ligand dissociation from the extracellular binding pocket was observed during the SMwST simulations (Figure S4).

We first analyzed the ligand-dependent effects on the global activation free-energy landscape of PAR1, as shown in Figure 2. Free energies were projected onto two widely used descriptors of class A GPCR activation: the aforementioned a100 index and the distance between residues Y290^5.58^ and Y371^7.53^ (Y–Y distance), which connects a conserved tyrosine in TM5 with a tyrosine in the DPxxY motif of TM7. According to the established interpretation of the a100 index, values greater than 55 correspond to fully active conformations, values between 0 and 55 represent intermediate states, and values below 0 indicate inactive conformations. Consistent with this interpretation, the Y–Y distance decreases progressively from approximately 20 Å in the inactive VPX-bound structure to roughly 7 Å in the active tethered agonist-bound structure.

**Figure 2:**
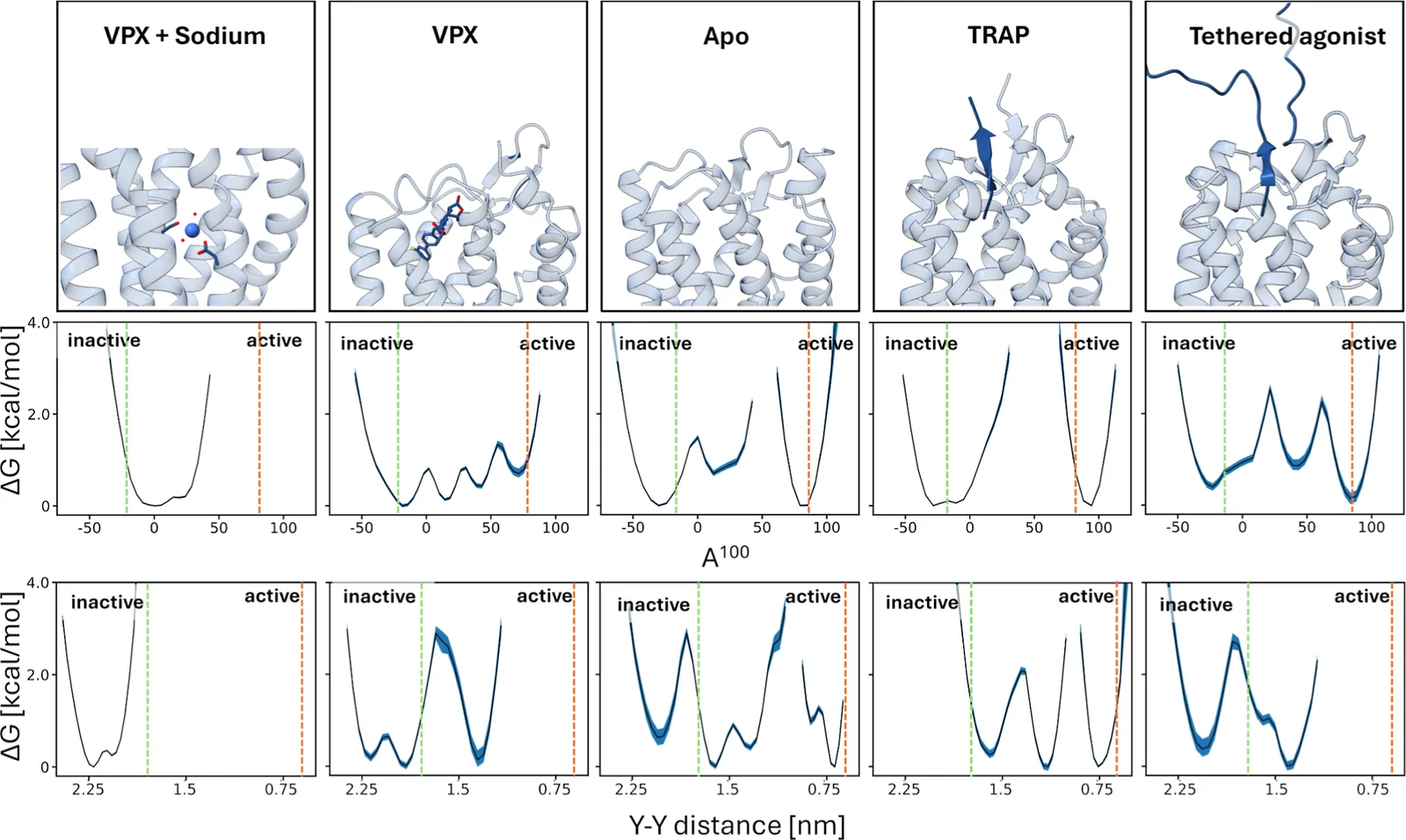
Ligand-dependent modulation of the PAR1 activation free-energy landscape. The ligand-bound PAR1 configurations investigated in this study are shown in the top row. Corresponding free-energy profiles are shown with the antagonist-bound systems on the left, the apo receptor in the center, and the agonist-bound systems on the right, and are projected onto the a100 activation index^30^ in the second row and onto the side-chain Cζ–Cζ distance between residues Y290^5.58^ and Y371^7.53^ (Y–Y distance) in the third row; the latter serving as a structural descriptor of conformational changes in the DPxxY motif of TM7. The blue transparent bands surrounding the free-energy profiles represent the 95% confidence intervals estimated by bootstrapping. Dashed lines indicate reference values derived from experimentally determined structures: green denotes the VPX-bound intermediate-state PAR1 crystal structure (PDB: 3VW7),^15^ and orange denotes the tethered agonist-bound active PAR1 cryo-EM structure (PDB: 8XOR).^17^ Discontinuous profile segments indicate regions with insufficient transition statistics. Quantitative free-energy differences should only be interpreted within continuously resolved regions of each profile.

The resulting free-energy landscapes largely recapitulate the expected qualitative behavior across the investigated receptor states (Figure 2). In the ligand-free (apo) condition, PAR1 samples a broad conformational ensemble encompassing inactive, intermediate, and active-like states, indicative of substantial intrinsic receptor flexibility. Discontinuities in the projected free-energy profile of the apo receptor and in some other systems arise from insufficient transition statistics between neighboring states, preventing estimation of free-energy values in the intervening regions of conformational space. Consequently, while the positions and characteristics of the sampled free-energy minima can be compared, the free-energies within the discontinuous regions cannot be resolved.

Binding of the antagonist VPX primarily restricts the local activation-associated rearrangement of the DPxxY motif, as reflected by the absence of short Y–Y distances. In contrast, the a100 projection indicates that the VPX-bound receptor can still populate multiple intermediate and active-like global conformations, although active-like states are increased in free energy relative to the inactive basin. Thus, VPX does not uniformly eliminate all active-like conformations across every projection but instead preferentially stabilizes conformations with an inactive-like DPxxY/TM7 arrangement. Consistent with this more heterogeneous behavior, the VPX-bound receptor retains notable conformational heterogeneity, with multiple intermediate free-energy minima apparent along the a100 coordinate. Strikingly, the combined presence of VPX and a sodium ion within the TM bundle eliminates these intermediate basins and strongly stabilizes the inactive receptor state. This condition yields a markedly simplified free-energy landscape that is shifted toward lower a100 values and larger Y–Y distances, consistent with previous reports of sodium-mediated stabilization of inactive class A GPCR conformations.^27^ For the VPX + Na^+^ system, active-state conformations were sampled rarely, resulting in insufficient transition statistics to estimate free-energy values in this region of the a100 projection.

In contrast, agonist binding shifts the projected free-energy profiles and underlying conformational ensemble toward more active conformational states. Binding of the TRAP peptide is associated with active-like conformations and reduces overall conformational heterogeneity, as evidenced by pronounced local minima at Y–Y distances of approximately 11 Å and 7.5 Å and by the absence of intermediate states in the a100 projection. However, a fully active conformation emerges as a clear global free-energy minimum in the a100 profile only in the presence of the complete tethered agonist, including the extracellular loop covalently linking the agonist to the receptor. Under these conditions, the active state is more strongly stabilized than the inactive state. This result indicates subtle yet energetically meaningful differences between PAR1 activation by soluble agonist peptides and activation by thrombin cleavage, despite engagement of the same binding-site residues. The distinct conformational ensembles sampled in these simulations may provide a structural basis for previously reported ligand-dependent differences in PAR1 downstream signaling and G_q_ coupling efficiency upon stimulation with agonist peptides versus thrombin.^31^

### Ligand Effects on the Minimum Free-Energy Pathway of PAR1 Activation

We computed free-energy landscapes along CVs describing TM6 outward displacement and TM7 inward shift to characterize probable activation pathways and compared these landscapes for the apo receptor and PAR1 bound to VPX + Na^+^ or the tethered agonist (Figure 3). In the apo system, two low free-energy regions are located near the conformations of the VPX-bound and tethered agonist-bound reference structures, corresponding to inactive-like and active-like regions of the landscape, respectively, along with one or two additional minima between these regions (Figure 3, middle column). This distribution indicates substantial intrinsic conformational flexibility, consistent with broad sampling of inactive-, intermediate-, and active-like conformations. Tracing the minimum free-energy pathway between inactive and active regions suggests that TM6 displacement, as reflected by increasing TM6–TM3/4 distances, precedes TM7 movement, which is captured by decreasing TM7–TM2/4 distances.

**Figure 3:**
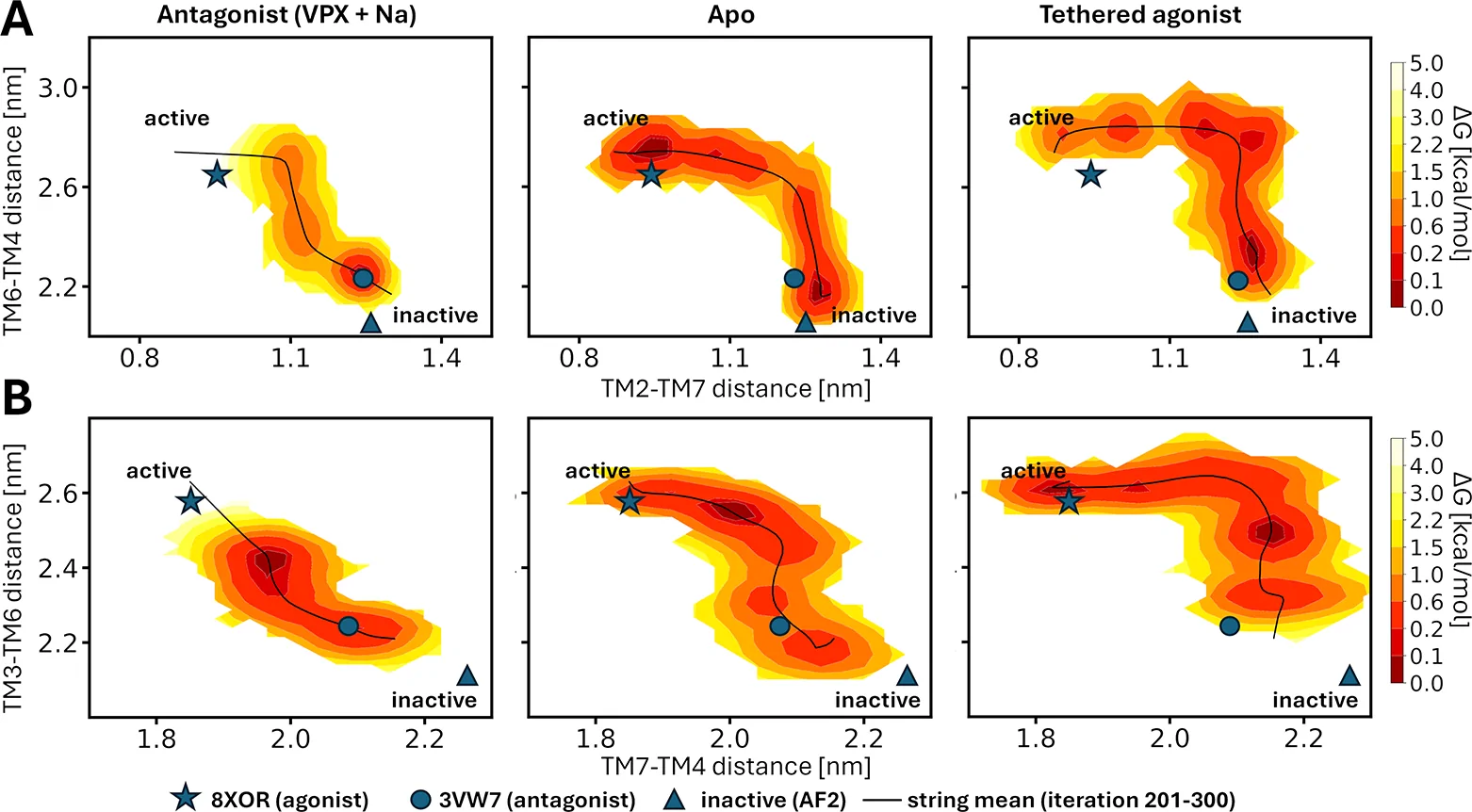
Ligand-dependent effects on the minimum free-energy pathway of PAR1 activation. Free-energy landscapes for the apo receptor and for PAR1 bound to VPX + Na^+^ or the tethered agonist are projected onto pairs of CVs describing TM6 outward displacement and TM7 inward shift during activation. The average string calculated from the final 100 iterations of the SMwST simulations is overlaid on each free-energy landscape. (A) TM6–TM4 distance versus TM7–TM2 distance. (B) TM6–TM3 distance versus TM7–TM4 distance. Low free-energy regions are shown in red, and high free-energy regions in light yellow; free energies are reported in kcal/mol. The positions of the three reference structures used to initialize the SMwST simulations (symbols defined at the bottom of the panels) are indicated on each landscape, and active and inactive regions are annotated.

In the presence of VPX and the allosteric sodium ion, the free-energy landscape becomes more constrained relative to the apo condition, indicating reduced conformational flexibility. The antagonist stabilizes inactive and intermediate states, as evidenced by energy minima located near the intermediate-state reference structure (PDB: 3VW7)^15^ midway between inactive and active regions (Figure 3, left column). In these projections, active-state conformations are not significantly populated in the VPX-bound simulation, as indicated by the absence of a corresponding energy minimum in the active region. Notably, the sequence of helix rearrangements differs from that of the apo receptor, with TM6 and TM7 movements occurring more synchronously rather than sequentially.

Binding of the tethered agonist further stabilizes active PAR1 conformations, most prominently in the TM6–TM3 versus TM7–TM4 projection (Figure 3B, right column), where the global minimum corresponds to the conformation with maximal TM6 outward displacement and TM7 inward shift, consistent with the PAR1-G_q_ structure (PDB: 8XOR).^17^ Although the inactive region is less populated in this projection, intermediate conformations remain accessible along the transition pathway, as indicated by two minima located midway between inactive and active regions. Similar to the apo landscape, the most probable activation pathway begins with outward displacement of TM6 (increasing TM6–TM3/4 distances), followed by the inward movement of TM7 (decreasing TM7–TM2/4 distances).

To assess whether the conformational space sampled by the SMwST simulations is consistent with unbiased receptor dynamics, we performed an additional 14.2 μs unbiased MD simulation starting from the equilibrated active-state PAR1 structure in the absence of ligands. During this simulation, PAR1 spontaneously transitioned toward inactive-like conformations, as reflected by a decrease in the a100 index, an increase in the Y–Y distance, an outward twist of TM7, and corresponding changes in TM6/TM7-related distances (Figure S5). Principal component analysis of the unbiased MD trajectory and the SMwST conformations showed substantial overlap in the space defined by the first two principal components, indicating that the selected CVs capture the major activation/deactivation-related conformational ensembles sampled without enhanced-sampling bias (Figure S5).

Collectively, our results show that ligand binding reshapes the PAR1 activation pathway by modulating helix rearrangement order and stabilizing distinct regions of the conformational landscape.

### Propagation and Coordination of Structural Rearrangements along the PAR1 Activation Pathway

Having established ligand-dependent modulation of PAR1 free-energy profiles, we next performed a detailed analysis of activation dynamics and the structural motifs that contribute to signal propagation within the receptor. Comparative analysis of conformations sampled along the equilibrated string trajectories enabled characterization of how local structural rearrangements associated with PAR1 activation propagate from the extracellular ligand-binding region toward the intracellular G-protein coupling interface. Principal component analysis of trajectory data from the final SMwST iteration, combined with back-projection-based feature importance analysis, identified ECL2, TM5, TM6, TM7, and ICL3 as the regions undergoing the most pronounced conformational changes during activation (Figure S6).

Based on these results, we selected a set of PAR1-specific inter-residue distance descriptors, together with established activation-related microswitches conserved among class A GPCRs, to quantitatively monitor activation-associated motions. The spatial locations of the selected descriptors and motifs are shown in Figure 4A, while their changes along the activation pathway, parametrized by the a100 activation index, is summarized in Figure 4B.

**Figure 4:**
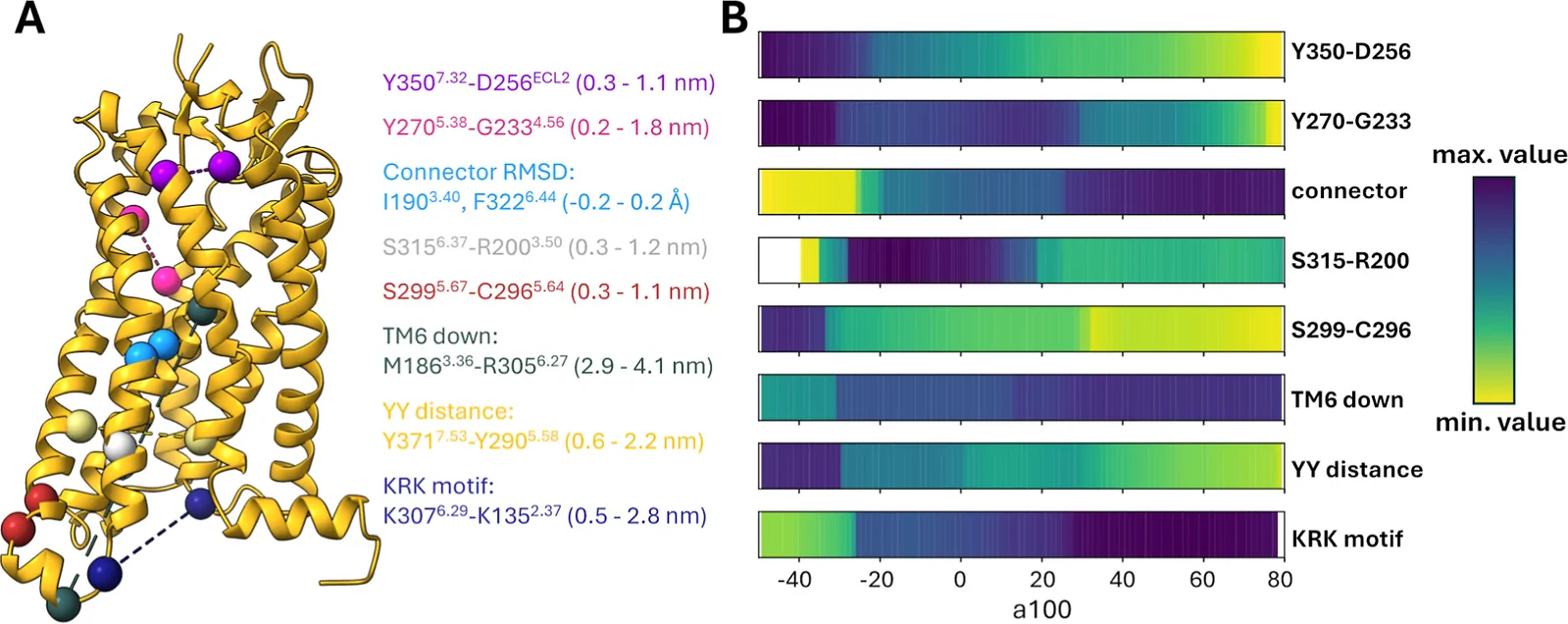
Structural descriptors of PAR1 activation and their progression along the activation pathway. (A) Spatial locations of selected structural motifs and inter-residue distances that undergo conformational rearrangements during PAR1 activation and are used as quantitative descriptors of the activation process. The legend reports the minimum and maximum values observed for each motif. (B) Activation trajectories of the selected descriptors, represented as color bars, where dark blue corresponds to the highest observed values (distance or rmsd), and yellow corresponds to the lowest values sampled in the apo receptor simulation. White regions indicate parts of the corresponding free-energy landscape that were not sampled. To relate these structural changes to overall receptor activation, values are plotted as a function of the a100 activation index, which serves as the *y*-axis coordinate. The progress bars were derived from 2D free-energy landscapes of the indicated observables projected against a100. A mean pathway was fitted to each landscape to define the progression coordinate, and the resulting curve was normalized using the minimum and maximum values shown in (A).

At the extracellular side of the receptor, ligand-induced compaction of the binding pocket and inward displacement of TM5 are reflected by a concerted decrease in the Y350^7.32^–D256^ECL2^ and Y270^5.38^–G233^4.56^ distances, respectively. These changes are coupled to the downward displacement of TM6, monitored by an increase in the M186^3.36^–R305^6.27^ distance. The resulting structural rearrangements are transmitted toward the intracellular side through the conserved connector motif, involving a coordinated side-chain rotamer switch of F322^6.44^ and I190^3.40^ located in TM6 and TM3, respectively.

At the intracellular face, activation is characterized by several coordinated changes: (i) a twist and inward displacement of TM7 at the DPxxY motif, evidenced by a decrease in the Y371^7.53^–Y290^5.58^ distance; (ii) disruption of the KRK motif, which coordinates a chloride ion in the inactive conformation but dissociates upon activation, as reflected by an increase in the K307^6.29^–K135^2.37^ distance; and (iii) reorganization and compaction of ICL3, captured by a shortening of the C296^5.64^–S299^5.67^ distance.

Beyond identifying these structural descriptors, we also examined the temporal ordering of conformational changes along the activation pathway. Projection of descriptor values onto the a100 reaction coordinate obtained from the final string of the apo receptor simulation (Figure 4B) reveals that early rearrangements (a100 ≈ −30 to −20) at the extracellular surface (e.g., Y350^7.32^–D256^ECL2^ distance) occur nearly concurrently with changes at the intracellular transducer-binding region (e.g., Y–Y distance and KRK motif). A second coordinated transition is observed at later stages of the pathway (a100 ≈ 20 to 30), preceding full receptor activation and consistent with the presence of a major intermediate state. Notably, several descriptors, including the Y350^7.32^–D256^ECL2^ distance and the DPxxY microswitch, exhibit more gradual changes along the reaction coordinate, suggesting the presence of multiple intermediate conformations rather than a single discrete intermediate.

The sequence of descriptor changes in the VPX + Na^+^ and tethered agonist-bound simulations is summarized in Figure S7. In the VPX + Na^+^ simulation, descriptors sample a narrower range of values, indicating reduced receptor flexibility and stabilization of inactive and intermediate states. Most descriptors remain inactive, including the Y350^7.32^–D256^ECL2^, Y270^5.38^–G233^4.56^, and Y371^7.53^–Y290^5.58^ distances, while TM6 downward movement and the KRK motif reflect only partial activation (Figure S7A). In contrast, the tethered agonist-bound simulation samples a similarly broad range of descriptor values as the apo receptor and follows a comparable ordering of changes along the a100 coordinate (Figure S7B). However, the Y350^7.32^–D256^ECL2^ distance and connector motif adopt even more active-like values, consistent with further stabilization of the active state.

Taken together, the data define the ordered propagation of activation-related conformational changes through PAR1 and show how different ligands affect this process. To further quantify these effects and to assess how ligand binding and G-protein coupling modulate these structural motifs, we next compared their conformational distributions across the different receptor–ligand systems investigated in this study.

### Free-Energy Analysis Reveals Ligand-Specific Modulation of PAR1 Activation Motifs

We computed free-energy landscapes as functions of the activation-related structural descriptors defined in Figure 4 to characterize how ligand binding modulates the propagation of conformational signals in PAR1. This analysis enabled a detailed assessment of how conformational changes initiated at the extracellular binding site are transmitted through the receptor and how these processes differ in the presence of VPX or the tethered agonist. Specifically, we evaluated ligand-dependent changes in TM7–ECL2 interaction, TM5 positioning, the connector motif, the DRF motif, ICL3, and the KRK motif. These descriptors were analyzed in conjunction with the PAR1-specific downward displacement of TM6, quantified by the M186^3.36^–R305^6.27^ distance, which provides a continuous metric that distinguishes inactive from active conformations over a range of approximately 1 nm (Figure 5). Using TM6 displacement as a reference coordinate indicates that the VPX-bound receptor does not substantially populate active-like states, as reflected by M186^3.36^–R305^6.27^ distances not exceeding ∼3.7 nm. In contrast, the ligand-free and tethered agonist-bound systems sample more downward-shifted TM6 conformations, reaching distances of up to ∼4.2 nm.

**Figure 5:**
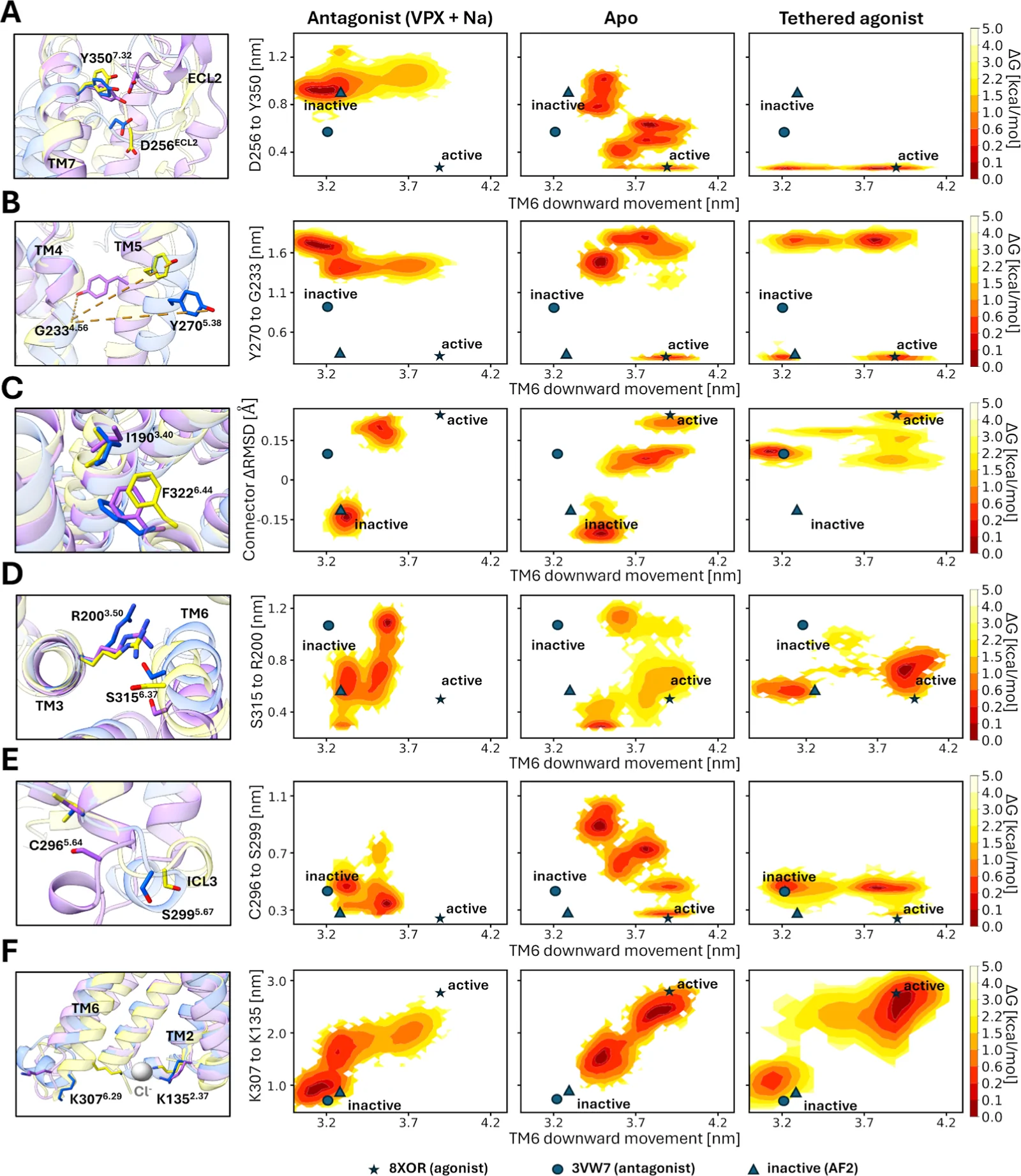
2D free-energy landscapes reveal stabilization of local PAR1 conformational states upon binding different ligands. Free-energy landscapes for the apo receptor and for PAR1 bound to VPX + Na^+^ or the tethered agonist are projected onto pairs of structural descriptors (defined in Figure 4) and correlated with the downward displacement of TM6, quantified by the M186^3.36^–R305^6.27^ distance. (A) Y350^7.32^–D256^ECL2^ distance, reporting agonist-dependent motions of ECL2. (B) Y270^5.38^–G233^4.56^ distance, reporting TM5 inward displacement. (C) Side-chain rmsd of the connector motif residues F322^6.44^ and I190^3.40^. (D) S315^6.37^–R200^3.50^ distance, describing rearrangements at the conserved DRF motif. (E) C296^5.64^–S299^5.67^ distance, reporting ICL3 reorganization. (F) K307^6.29^–K135^2.37^ distance, reflecting the chloride ion coordination state of the KRK motif and the associated outward displacement of TM6. Low free-energy regions are shown in red, while high free-energy regions are shown in light yellow; free energies are reported in kcal/mol. The positions of the three reference structures used to initialize the SMwST simulations (symbols defined at the bottom of the panels) are indicated on each landscape, and active and inactive regions are annotated. Structural snapshots on the left illustrate representative conformations of the descriptors in the active state (PDB: 8XOR, purple), inactive state (PDB: 3VW7, yellow), and an intermediate conformation (blue) sampled from the converged string approximately midway between the active and inactive regions.

At the extracellular interface, residue Y350^7.32^ plays a key role in activation. In the active structure, Y350^7.32^ directly contacts the tethered agonist and stabilizes ECL2 via hydrogen bonding with D256^ECL2^ (Figure 5A). In the ligand-free receptor, this interaction is transient, resulting in increased ECL2 flexibility reflected by multiple local minima along the Y350^7.32^–D256^ECL2^ coordinate. VPX binding restricts ECL2 dynamics while maintaining an inactive receptor conformation, in which the Y350^7.32^–D256^ECL2^ hydrogen bond is not observed. In contrast, tethered agonist engagement promotes a downward displacement of Y350^7.32^, stabilizing this interaction and potentially facilitating a concerted inward shift of TM7.

Activation further involves rearrangements of aromatic residues in TM5, particularly F271^5.39^ and Y270^5.38^, accompanied by a displacement and modest rotation of TM5 toward TM4, monitored by the Y270^5.38^–G233^4.56^ distance (Figure 5B). In the active conformation, these residues form a hydrogen bond, whereas in inactive states, they are separated by more than 1 nm. This interaction is absent in the VPX-bound system but is sampled in both active and inactive ensembles of the tethered agonist-bound and apo receptors, as indicated by minima at both short and long distances and by the absence of stable intermediate configurations. This behavior suggests a direct influence of tethered agonist engagement on TM5 positioning, potentially mediated by stabilization of ECL2. Notably, the inactive-state AlphaFold2 model also exhibits this interaction, raising the possibility that the Y270^5.38^–G233^4.56^ hydrogen bond may be modulated by VPX binding.

Within the TM core, the conserved connector motif formed by F322^6.44^ and I190^3.40^ has been proposed to mediate signal transmission between the orthosteric site and the intracellular interface. Connector dynamics was quantified by the difference between the rmsds of these residues relative to their positions in the active reference structure (PDB: 8XOR)^17^ and inactive-state AlphaFold2 model (Figure 5C). Across ligand conditions, three dominant connector states were observed: active (>+0.15 Å), inactive (∼−0.15 Å), and intermediate (0–0.15 Å). The intermediate state resembles the VPX-bound conformation, while active and inactive states correspond to the motif conformations in the tethered agonist-bound structure and the ligand-free AlphaFold2 model, respectively. The intermediate configuration is most prominently sampled in agonist-bound simulations, although it remains accessible in ligand-free and VPX-bound systems as well. These observations suggest that the connector motif can transiently adopt active-like arrangements even in the presence of VPX. As expected, the inactive connector configuration is absent in tethered agonist-bound simulations but present in VPX-bound and ligand-free systems.

Closer to the transducer-binding interface, two interaction motifs exhibit pronounced dynamics despite not being immediately evident from static structural comparisons. One involves S315^6.37^ in TM6, which can form a hydrogen bond with R200^3.50^ in the conserved DRF motif, stabilizing inactive conformations by restricting outward TM6 displacement (Figure 5D). This interaction is observed in VPX-bound and ligand-free simulations, but it is absent in tethered agonist-bound systems, as indicated by the lack of a short-distance peak in the corresponding free-energy landscape. The second motif involves the C296^5.64^–S299^5.67^ pair in ICL3, reporting on ICL3 conformational variability. TM6 downward displacement during activation alters ICL3 geometry, enabling formation of a stable hydrogen bond between the S299^5.67^ side chain and the backbone of C296^5.64^ (Figure 5E). This interaction is present in the tethered agonist-bound structure and simulations. While the C296^5.64^–S299^5.67^ distance increases only slightly in the VPX-bound system, the ligand-free receptor samples multiple minima at larger distances, indicating enhanced ICL3 flexibility in the absence of extracellular ligand or intracellular G protein. This observation is consistent with experimental and mutational studies implicating ICL3 in PAR1 coupling to G proteins,^17^ an aspect examined further in the following section.

At the G-protein-binding interface, the VPX-bound structure (PDB: 3VW7)^15^ reveals a chloride ion coordinated by K135^2.37^, R200^3.50^, and K307^6.29^ (Figure 1). This arrangement may sterically or electrostatically hinder G-protein engagement and could functionally substitute for the canonical ionic lock motif present in many class A GPCRs but absent in PAR1. Although chloride ions are not generally implicated in GPCR activation, and are often attributed to crystallization conditions,^32^ several observations suggest a more specific role in PAR1. The coordinating residues undergo substantial rearrangements between activation states consistent with this observation, alanine mutagenesis of the KRK motif residues has previously been shown to impair both G_q_- and G_i_-mediated signaling of PAR1, highlighting their functional importance.^17^

In the VPX-bound system, the K135^2.37^–K307^6.29^ distance remains short (∼1 nm), stabilizing an inactive-like configuration, whereas tethered agonist-bound simulations preferentially sample larger separations (2.5–3.0 nm) characteristic of active-like states (Figure 5E). The ligand-free system samples two minima at intermediate distances. Notably, chloride coordination by the KRK motif is also observed in simulations in which no ion was initially placed at this site, with R214^34.57^ contributing as an additional coordination partner (Figure S8). Together, these results suggest that chloride coordination is associated with inactive-state conformations of PAR1 and may contribute to local stabilization of the positively charged KRK/DRF region through electrostatic screening. However, the present simulations are not enough to establish a causal functional role for chloride binding in PAR1 activation or signaling, and additional experiments would be required to determine whether chloride acts as a specific allosteric regulator rather than as a stabilizing electrostatic interaction.

Collectively, our free-energy analysis indicates that VPX stabilizes inactive PAR1 by restricting TM5 and TM6 movements, maintaining inactive conformations of ECL2 and the connector motif, and preserving short KRK motif distances. In contrast, the tethered agonist stabilizes the active state by promoting TM5 and TM6 rearrangements, reinforcing the Y350^7.32^–D256^ECL2^ interaction, and driving ICL3 reorganization, together with increased separation of the KRK motif. These ligand-specific effects delineate a continuous structural pathway linking extracellular ligand engagement to intracellular DRF, ICL3, and KRK rearrangements that govern G-protein coupling.

### Gα_q_ Binding Constrains TM6 Dynamics and Stabilizes the Active-State Conformation of PAR1

Beyond orthosteric ligand binding, an important aspect of GPCR activation is the allosteric coupling between the receptor and intracellular transducers. Biochemical and biophysical studies of receptors such as β_2_AR and A_2_AR have shown that G-protein binding to the agonist-receptor complex shifts the conformational equilibrium toward active states and enhances agonist affinity by forming the ternary complex.^33–36^ Structural and computational investigations further demonstrate that transducer engagement stabilizes hallmark active-state features, including TM6 displacement and closure of the ligand-binding pocket, thereby propagating conformational changes from the cytosolic interface to the orthosteric site via long-range coupling.^34–36^ For PAR1, its high-resolution cryo-EM structure in complex with Gα_q_ provides a well-defined structural framework for investigating transducer-dependent stabilization of the active receptor state.^17^

To examine the influence of Gα_q_-protein binding on the PAR1 conformational energy landscape, we constructed a model of the TRAP–PAR1 complex bound to nucleotide-free Gα_q_ subunit and performed an additional string simulation covering the active portion of the pathway sampled in the TRAP-bound system. The use of a nucleotide-free Gα_q_ subunit as computationally tractable model of the receptor-transducer interface follows previous MD simulation studies,^37–39^ as the majority of direct receptor-G protein contacts are mediated through Gα, whereas inclusion of the Gβγ subunits would substantially increase the computational costs. Simulations including the full heterotrimeric G protein and alternative nucleotide-bound states may further modulate receptor dynamics and will be important directions for future investigation.

Consistent with previous structural and mutational analyses of the PAR1-Gα_q_ interface,^17^ our simulations confirm an important role of ICL2 and ICL3 in Gα_q_ engagement (Figure 6A). In ICL3, S306^6.28^ forms polar contacts with Q365 and a hydrogen bond with D361 on the Gα_q_ α5 helix. Stable interactions were observed for several ICL2 residues, including R214^34.57^, which forms a salt bridge with E370 and a hydrogen bond with Y371 on the Gα_q_ α5 helix. Thus, beyond its role in chloride coordination, R214^34.57^ may contribute directly to Gα_q_ coupling, a prediction that we tested experimentally using a bioluminescence resonance energy transfer (BRET)-based Gα_q_βγ dissociation assay^40^ described in the following section. Additional contacts involve the tip of the Gα_q_ α5 helix, including a polar interaction with M141^2.43^ in TM2 and a transient interaction with R310^6.32^, which we likewise evaluate experimentally.

**Figure 6:**
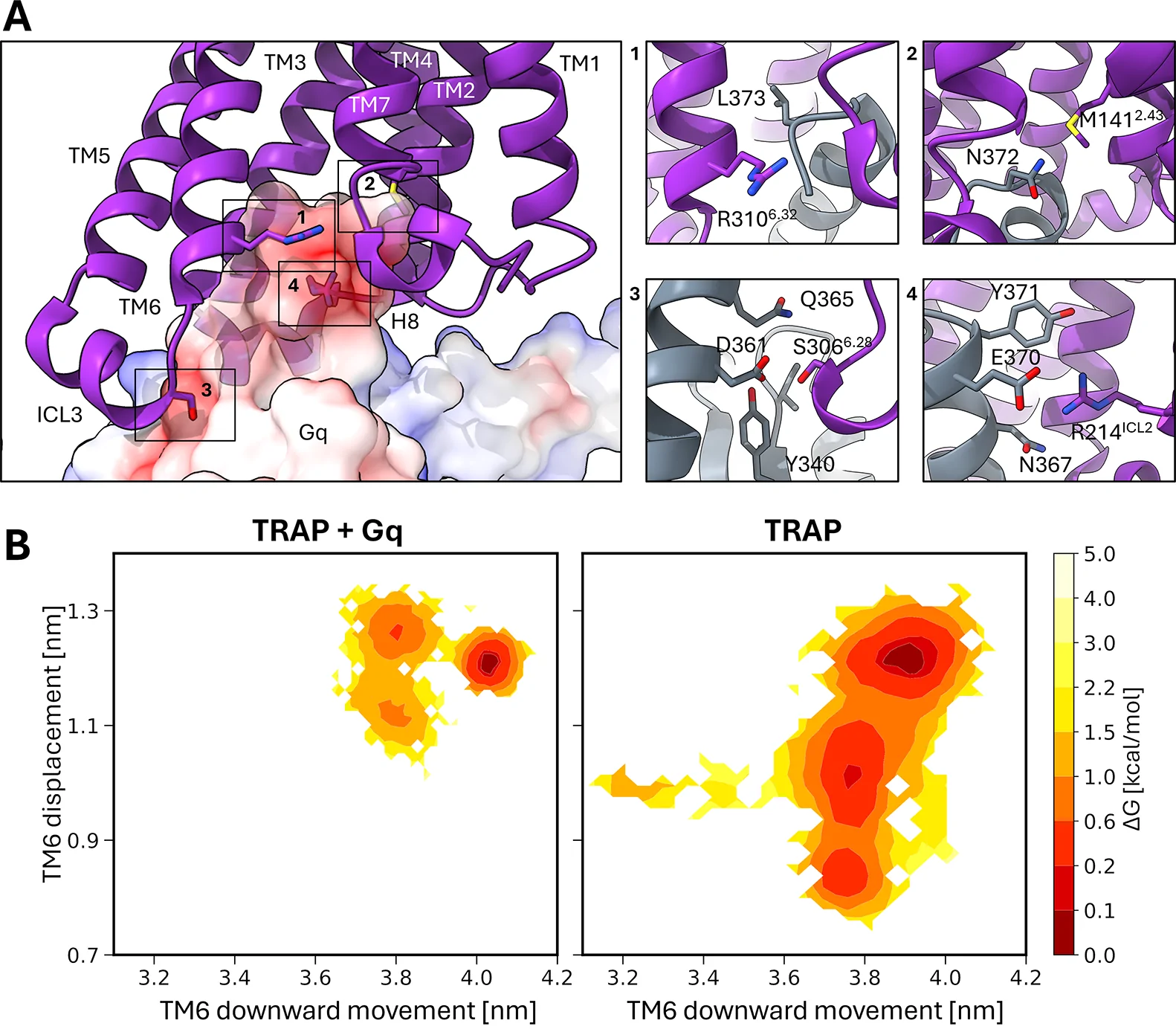
Gα_q_ binding stabilizes an outward- and downward-displaced TM6 conformation in active PAR1. (A) Engagement of the Gα_q_ subunit with the cytosolic interface of PAR1. PAR1 is shown as a cartoon representation in purple, while Gα_q_ is depicted either as an electrostatic surface (left) or as a cartoon model in gray (right). Insets highlight selected PAR1 residues at the receptor-Gα_q_ interface and their interactions with the Gα_q_ subunit. (B) 2D free-energy landscapes illustrate the effect of Gα_q_ binding on the PAR1 conformational ensemble, projected onto descriptors of TM6 downward displacement and outward movement, revealing stabilization of the active-state TM6 configuration.

Analysis of the resulting free-energy landscapes for the TRAP-bound and TRAP + Gα_q_ systems, projected onto descriptors of TM6 downward and outward displacement (Figure 6B), reveals a pronounced effect of transducer binding. In the presence of Gα_q_, TM6 flexibility is reduced, and the accessible conformational space is constrained, most notably through stabilization of a larger and more confined outward displacement. A modest increase in TM6 downward displacement is also observed, consistent with a shift in the free-energy minimum toward more active-like conformations upon G-protein engagement.

Collectively, these results demonstrate that Gα_q_ binding allosterically stabilizes active PAR1 conformations by constraining TM6 dynamics and reinforcing intracellular coupling interfaces.

### Experimental Validation of Computationally Identified PAR1 Activation and G-Protein Binding Sites

To experimentally assess the functional relevance of residues predicted by the simulations to influence PAR1 activation and subsequent Gα_q_ engagement signal, selected positions were mutated to alanine and evaluated using a BRET-based Gα_q_βγ dissociation assay (G-CASE).^40^

Based on the free-energy analysis (Figure 5), residues Y270^5.38^, S299^5.67^, S315^6.37^, and Y350^7.32^ were selected for mutagenesis due to their predicted roles in propagating activation-associated conformational changes. In addition, residues M141^2.43^, R214^4.57^, S306^6.28^, and R310^6.32^ were targeted based on their involvement in the simulated PAR1-Gα_q_ interface (Figure 6). All mutants were confirmed to show similar cell surface expression levels as the WT PAR1 (Figure S9). To report both statistical significance and the magnitude of the mutational effects, shifts in efficacy (*E*_max_) and potency (pEC_50_), together with their 95% confidence intervals and statistical comparisons with WT PAR1, are provided in Table S2.

The Y350^7.32^A mutation produced a significant reduction in PAR1-mediated Gα_q_ activation (*E*_max_) by TRAP (*p* < 0.0001, Figure 7A and Table S2), consistent with its structural role in stabilizing the active conformation and with previous mutational studies.^17^ Mutations S299^5.67^A and S315^6.37^A also significantly reduced the maximal response to TRAP (*p* < 0.0001 and *p* = 0.0462, respectively, Figure 7A and Table S2), consistent with simulation results indicating roles of these residues in modulating ICL3 conformation and in forming interactions with the DRF motif residue R200^3.50^. In addition, S315^6.37^A significantly reduced receptor potency (pEC_50_) to 5.42 compared to 7.57 for WT PAR1 (*p* < 0.0001, Table S2).

**Figure 7:**
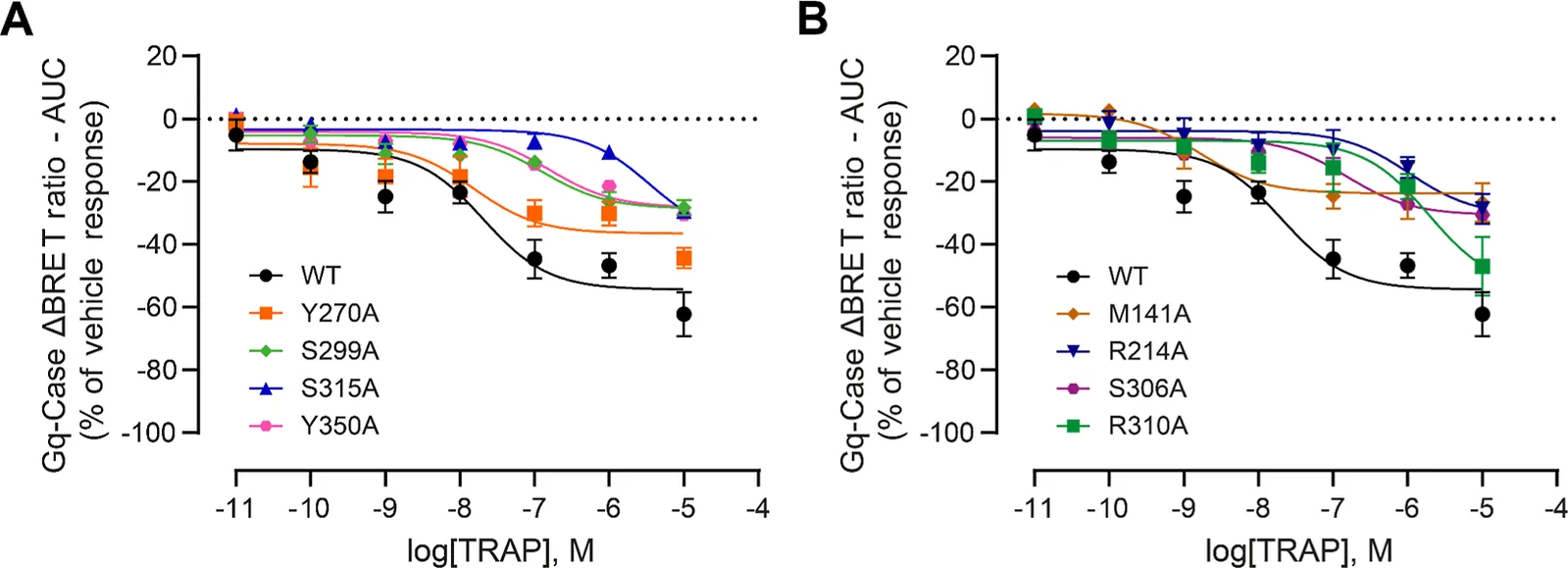
Effects of PAR1 alanine substitutions on G_q_ coupling efficiency. Concentration–response curves show TRAP-induced G_q_ activation for PAR1 alanine mutants measured using a Gα_q_βγ dissociation BRET assay. (A) Mutations of residues implicated in tethered agonist binding and in the propagation of activation-associated conformational changes along the receptor (structural context shown in Figure 5). (B) Mutations of residues located at the G_q_-binding interface (structural context shown in Figure 6). Data represent mean ± SEM from *n* = 3 independent biological replicates. Data are normalized to the buffer-only (vehicle-treated) condition. Curves represent the fit of a three-parameter logistic equation. *E*_max_ and pEC_50_ values of BRET signals were compared to WT PAR1 using a one-way ANOVA followed by Fisher’s LSD multiple comparisons test. *P*-values for comparisons with WT PAR1 are provided in Table S2.

The Y270^5.38^A mutation, selected based on its interaction with G233^4.56^, resulted in reduced G_q_ activation (*p* = 0.0296, Figure 7A). Alanine mutagenesis has previously revealed the adjacent residue F271^5.39^ to be critical for PAR1 activation,^17^ and conformational changes involving Y270^5.38^ may accompany those of F271^5.39^.

Alanine substitutions at residues predicted to participate in G-protein binding also reduced PAR1-mediated G_q_ dissociation (Figure 7B and Table S2). Mutations M141^2.43^A and S306^6.28^A produced the largest decreases in *E*_max_ upon TRAP stimulation (both *p* < 0.0001), consistent with their direct interactions with the Gα_q_ α5 helix and with prior mutational analyses.^17^ M141^2.43^A additionally exhibited a significant increase in receptor potency (pEC_50_, *p* < 0.0001, Table S2). The pronounced reduction in *E*_max_ together with the altered pEC_50_ observed for M141^2.43^A may reflect impaired stabilization of a signaling-competent PAR1-Gα_q_ complex, although the present assay cannot distinguish whether this arises from altered receptor activation, Gα_q_ engagement, or changes in apparent agonist potency. The R214^4.57^A mutation also significantly impaired *E*_max_ (*p* = 0.0219) and pEC_50_ (*p* = 0.0001), whereas R310^6.32^A exhibited an *E*_max_ comparable to the WT but lower potency (*p* = 0.0040, Figure 7B and Table S2).

Overall, these experimental results support the functional relevance of multiple interaction motifs identified in the MD simulations and demonstrate the utility of integrating simulation-derived hypotheses with targeted mutagenesis to elucidate GPCR activation mechanisms and identify residues critical for receptor-G protein coupling.

## Conclusion

This study provides an atomically detailed characterization of PAR1 activation dynamics that complements existing crystal and cryo-EM structures by elucidating how extracellular ligands and intracellular transducers reshape the receptor’s conformational energy landscape and microswitch network. By integrating enhanced-sampling MD simulations with experimental validation, we investigate how ligand identity and G-protein engagement influence the stability and coupling of activation-related structural motifs that underlie PAR1 signaling.

Our simulations show that PAR1 exhibits substantial intrinsic flexibility, sampling inactive, intermediate, and active states under the apo condition. Antagonist binding (VPX + Na^+^) constrains the conformational landscape and stabilizes inactive states by restricting TM5 and TM6 rearrangements, maintaining inactive ECL2 and connector conformations, and preserving a compact KRK motif. In contrast, agonist binding shifts the equilibrium toward active conformations, with the full tethered agonist most strongly stabilizing TM5 and TM6 rearrangements, TM7 movement, and ICL3 reorganization. Free-energy analyses further reveal that ligand binding modulates the minimum free-energy pathway, altering the order and coupling of TM6 and TM7 motions and reshaping intermediate-state populations.

At the level of signal propagation, our results identify a continuous structural pathway linking extracellular ligand engagement to intracellular coupling interfaces. Key nodes in this pathway include TM5 repositioning, rearrangements of the connector and DRF motif, KRK motif dynamics, and ICL3 reorganization. Notably, chloride coordination at the KRK motif is consistently associated with inactive-state conformations in our simulations. While this observation raises the possibility that chloride ions may contribute to receptor stabilization, direct experimental evidence for a functional role is currently lacking. Experimental alanine mutagenesis and Gα_q_βγ-dissociation BRET assays support the functional relevance of residues predicted to mediate activation and G-protein coupling, underscoring the predictive value of the simulations.

Incorporation of Gα_q_ into the simulations reveals that transducer binding constrains TM6 dynamics and stabilizes active-like TM6 conformations, while reinforcing intracellular interaction networks involving ICL2 and ICL3. These findings support a reciprocal coupling mechanism in which G-protein engagement not only recognizes active receptor conformations but also stabilizes and refines them, thereby reshaping the conformational ensemble and potentially modulating agonist efficacy.

While the simulations employed here explore a wide range of conformations, they rely on predefined CVs and finite string resolution, which may bias the explored pathways and overlook slower orthogonal motions. Force-field limitations, the use of an AlphaFold-modeled inactive structure, and the absence of full cellular context, including membrane heterogeneity, additional post-translational modifications, and competing transducers, may influence the quantitative features of the free-energy landscapes. Moreover, the Gα_q_-bound simulations were performed in the nucleotide-free state and with a monomeric Gα_q_ subunit, which likely does not fully capture the full spectrum of transducer-dependent conformations.

Looking ahead, extending this framework to additional PAR1 ligands, such as parmodulins, pepducins, and protease-specific tethered agonists, will help clarify how distinct extracellular cues bias PAR1 signaling.^9^ Simulations incorporating alternative transducers (Gα_i_, Gα_12/13_), heterotrimeric GTP-bound G proteins and β-arrestins, together with longer-time scale sampling and complementary structural experiments, will be essential to map transducer-specific conformational ensembles and validate predicted intermediate states.

From a broader perspective, a deeper understanding of how different ligands shape the PAR1 conformational landscape may inform the rational design of pathway-selective therapeutics. For example, parmodulins that bias PAR1 signaling toward cytoprotective pathways, or next-generation antithrombotic agents derived from VPX scaffolds with reduced bleeding risk,^9^ could benefit from mechanistic insight into how they differentially stabilize receptor conformations and coupling interfaces, ultimately enabling the development of PAR1-targeting drugs with improved safety and efficacy profiles.

## Methods

### Structure Preparation

The following PAR1 structures were used as starting structures for performing MD simulations: (i) a PAR1 crystal structure bound to the antagonist VPX (PDB: 3VW7),^15^ which represents an intermediate receptor state, (ii) the open-state PAR1 structure in complex with the TRAP peptide ligand (SFLLRN) and Gα_q_ (PDB: 8XOR),^17^ and (iii) an AlphaFold2-predicted PAR1 structure provided by GPCRdb,^16^ which adopts a more strongly inactive conformation according to the widely used a100 activation index.^30^ The use of the AlphaFold2 model as the inactive end point for the simulations was further supported by comparison with available experimental structures and activation-state descriptors (Figure S1). In particular, the AlphaFold2 model adopted more inactive conformations of key activation motifs, including TM6, the connector motif, and the DPxxY motif, than the VPX-bound structure (PDB: 3VW7). In addition, comparison with active and inactive structures of the related apelin receptor and neurotensin receptor showed that the AlphaFold2 model displayed greater similarity to inactive-state reference structures, whereas the VPX-bound crystal structure exhibited a more intermediate-state character.

Residue protonation states were assigned using PROPKA,^41^ which indicated that either D367^7.49^ or D148^2.50^ in PAR1, located in close spatial proximity, should be protonated. Based on this result and in agreement with previous findings,^42^ the side chain of D148^2.50^ was protonated in all MD systems.

Following the procedure described by Alfonso-Prieto and Capelli^43^ the inactive PAR1 model obtained from GPCRdb was further stabilized by adding a sodium ion at a position inferred from the PAR1 crystal structure, where the ion is coordinated by residues D148^2.50^, D367^7.49^, S363^7.45^, and S189^3.39^. To generate an additional model of thrombin-cleaved PAR1, in which the tethered agonist is covalently linked to the receptor via the TM1-preceding loop, AlphaFold2-Multimer^44^ was employed, using the PAR1-Gα_q_ structure (PDB: 8XOR)^17^ as an input template. Missing structural elements were also modeled with AlphaFold2-Multimer during this step, except for the unstructured PAR1 C-terminus (residues ≥ 392), which was removed due to low confidence, as indicated by its pLDDT score in the AlphaFold2-Multimer prediction.

### MD System Preparation and Equilibration

A total of six MD systems were prepared using the CHARMM-GUI web interface:^45,46^ (i) ligand-free PAR1 (apo), (ii) PAR1 in complex with the agonist TRAP peptide, (iii) PAR1 in complex with its thrombin-generated tethered agonist, (iv) PAR1 in complex with the antagonist VPX, (v) PAR1 bound to VPX with an additional sodium ion located inside the TM bundle, and (vi) PAR1 in complex with the TRAP peptide and the Gα_q_ subunit in the nucleotide-free state.

All protein termini were assigned neutral capping groups (i.e., NH_2_ and COOH), except for the N-terminus of the tethered agonist and the N- and C-termini of the TRAP peptide, which were modeled in their ionized states (i.e., NH_3_^+^ and COO^–^). Additional post-translational modifications included a disulfide bond between C175^3.25^ and C254^ECL2^, as well as palmitoylation of C387^8.59^ and C388^8.60^ at the receptor C-terminus.

All structures were aligned to the membrane reference frame using the OPM Web server^47^ and subsequently embedded in a phosphatidylcholine (POPC) lipid bilayer consisting of approximately 90 POPC molecules per leaflet. Systems were solvated with approximately 80 TIP3P water molecules per lipid and neutralized with NaCl to a final concentration of 0.15 M. MD system setup is summarized in Table S3.

The charmm36m force field^48^ was used to parametrize all protein, lipid, and ion components, except for the small-molecule antagonist VPX, which was parametrized using the CGenFF Web server.^49^

Following energy minimization and equilibration using the standard CHARMM-GUI protocol, the final equilibration phase was extended to 100 ns, and an additional 100 ns equilibration was performed with a harmonic restraint of 5 kJ mol^–1^ nm^–2^ applied to the protein backbone to prevent system destabilization. Production runs were carried out using GROMACS (version 2021)^50^ patched with PLUMED (version 2.7.1)^51^ in the *NPT* ensemble, with the temperature maintained at 310.15 K and the pressure at 1 bar using the Nosé–Hoover thermostat and a semi-isotropic barostat, respectively. An integration time step of 2 fs was used throughout the simulations.

### Targeted MD

To generate the initial string of conformations for subsequent string method with swarms of trajectories (SMwST) simulations, ensembles of PAR1 conformations evenly distributed between the active and inactive states were produced using targeted molecular dynamics (tMD). tMD employed a biasing force based on the root-mean-square deviation (rmsd) to a defined target conformation. The resulting tMD trajectories were used solely to initialize the string, which was subsequently refined through iterative SMwST optimization.

For all MD systems except those with VPX bound, the initial structure for tMD corresponded to the active-state PAR1 structure in complex with the tethered agonist and Gα_q_ (PDB: 8XOR),^17^ prepared as described above. tMD was performed in two sequential steps: first, the VPX-bound PAR1 crystal structure (PDB: 3VW7)^15^ was used as the target conformation; second, the inactive AlphaFold2-generated PAR1 structure obtained from GPCRdb was employed as the final target. In contrast, the initial string for the VPX-bound systems was generated by starting from the VPX-bound crystal structure and performing two independent tMD simulations: one targeting the active-state PAR1 structure (PDB: 8XOR)^17^ and the other targeting the inactive AlphaFold2-generated model.

To prevent global translations and rotations of the receptor from contributing to the rmsd calculation, PAR1 was aligned at each MD step to the target structure using TM helices TM1–TM4, which undergo only minimal conformational changes during receptor activation. The rmsd between the aligned structures was then computed over all receptor heavy atoms, excluding the PAR1 N- and C-termini and ECL2; ECL2 was omitted to prevent bias-induced ligand unbinding in this region. Based on the instantaneous rmsd value, a harmonic biasing potential with a force constant of 10,000 kJ·mol^–1^·nm^–2^ was applied to all included heavy atoms. Each tMD simulation was run for 250 ns using GROMACS (version 2021)^50^ patched with PLUMED (version 2.7.1).^51^

### String Method with Swarms of Trajectories (SMwST)

The SMwST method^52^ iteratively refines an initial transition path between two metastable states to identify the most probable transition pathway. Refinement is performed using a set of collective variables (CVs) that are selected to capture the slow, functionally relevant motions connecting the metastable states. To sample conformational transitions of the PAR1 receptor, we employed a set of CVs previously described by Fleetwood et al.,^28^ which reliably discriminate between active and inactive GPCR conformations, together with the a100 activation index for additional characterization.^30^ The CVs used in this study are listed in Table S1. We verified that this combined set of metrics effectively distinguishes active, intermediate, and inactive PAR1 structures (Figure S2).

The string consisted of 42 images, corresponding to independent copies of the MD system. The two end point images, representing the active and inactive PAR1 conformations, respectively, were held fixed, while the remaining 40 intermediate images were allowed to evolve during string refinement. For each of the six MD systems described above (PAR1 apo, PAR1 + TRAP, PAR1 + tether agonist, PAR1 + VPX, PAR1 + VPX + Na^+^, and PAR1 + TRAP + Gα_q_), 40 initial conformations were selected from the preceding tMD simulations such that they were evenly distributed in both CV space (Table S1) and a100 space, spanning active, intermediate, and inactive states. These conformations served as initial strings for the SMwST simulations.

Iterative string refinement was carried out according to the workflow described below using Python scripts provided by the Delemotte lab (https://github.com/delemottelab/stringmethod-swarms-trajectories):^28^

(1) String reparameterization. To ensure equal weighting of all CVs, the string was normalized such that each CV ranged from 0 to 1, and subsequently reparameterized to enforce equal arc length between consecutive images.
(2) Restrained simulations. Starting from the output conformations of the previous string iteration, short MD simulations with an additional bias applied to the CVs were performed to generate updated image conformations along the newly parametrized string. The simulation length was 4 ns for the initial string and 30 ps for all subsequent string iterations.
(3) Swarm simulations. For each image along the string, a swarm of 30 unbiased MD simulations, each 10 ps in length, was launched.
(4) Computation of the next string. After normalization, the mean drift of each swarm in CV space was calculated and used to update the image positions. The updated positions of all images were then assembled to generate the string for the next iteration.

Convergence between consecutive strings was assessed by computing the Frobenius norm, which served as a quantitative measure of the distance between two strings.

Following the procedure described by Fleetwood et al.,^28^ exchange of conformations between images along the string was enabled. Specifically, after 10 SMwST iterations, the restrained simulations (step 2) were not necessarily initialized from the final output conformation of the immediately preceding iteration. Instead, the conformation closest to the updated string image was identified from the pool of output conformations generated across recent iterations, based on the Frobenius norm, and used as the starting structure for the subsequent restrained simulation.

All MD simulations within the SMwST protocol were performed using GROMACS (version 2021)^50^ patched with PLUMED (version 2.7.1).^51^ The total simulation time of each SMwST simulation for PAR1 with the different extracellular ligands was 3.4 μs (Table S3), while for the PAR1-Gα_q_ system it was 1.7 μs.

### Free-Energy Calculation

Following the workflow described by Fleetwood et al.,^28^ the free-energy landscape of the system was calculated along selected CVs by analyzing the dynamics of swarm trajectories after string convergence and equilibration of the MD system. Briefly, the CV space was discretized into finite states (bins), and the number of transitions between states was counted from the swarm trajectories. From these counts, a transition probability matrix T was constructed, where the transition probability T_ij_ between states i and j was calculated as the number of observed transitions N_ij_ divided by the total number of transitions *N_i_*_*k*_ originating from state *i*

1 $$T_{ij}=\frac{N_{ij}}{\sum_{k}N_{ik}}$$

The stationary distribution ρ, corresponding to the eigenvector of the transition matrix, was then used to compute the free energy Δ*G* of each state using the Boltzmann relation

2 $$\Delta G={-k}_{B}\cdot T\cdot \log\left(\rho_{i}\right)$$

For the free-energy analysis, swarm trajectories from the final 100 iterations of the SMwST simulations were used, and the selected CVs were computed using the mdTraj package.^53^ Transition probability matrices, free-energy surfaces, and error estimates obtained via Monte Carlo bootstrapping were calculated using the pyEMMA package.^54^

### Principal Component Analysis

A subset of 1320 PAR1 conformations sampled from the full string of the final 100 iterations of the SMwST simulations was used to calculate inter-residue Cα–Cα distances using the MDAnalysis package.^55^ This resulting distance matrix was then subjected to unsupervised feature extraction using principal component analysis (PCA) as implemented in the Demystify software.^56^ By weighting the principal components by their corresponding eigenvalues and back-projecting them onto the original Cα–Cα distance features, the contribution of each input feature to the total variance—and thus its relative importance—could be determined.

### Cell Culture and PAR1 CRISPR/Cas9 KO

HEK293T cells with PAR1 knock out (KO) used for transient expression of the PAR1 mutants and G protein assay were grown in DMEM-GlutaMAX, supplemented with 10% fetal bovine serum, streptomycin (0.1 mg/mL), and penicillin (100 U/ml) at 37 °C with 5% CO_2_. PAR1 KO was performed with CRISPR/Cas9 KO plasmids (h) (sc-400556-KO-2, Santa Cruz) according to manufacturer instructions and verified by surface protein expression.

### PAR1 Site-Directed Mutagenesis and G_q_-CASE BRET Assays

Site-directed mutagenesis of human PAR1 was performed to introduce single-amino acid substitutions at selected positions. Mutations were generated using the Q5 Site-Directed Mutagenesis Kit (E0554S, NEB, Germany) according to the standard protocol with sequence-specific primers (Table S4) and verified by Sanger sequencing. Wild-type and mutant PAR1 constructs were expressed in HEK293T cells using pCMV3-C-FLAG expression vectors (HG13535-CF, Sino Biological, Germany).

For signaling measurements, cells were transiently cotransfected with 500 ng plasmids encoding PAR1 variants together with 500 ng the G_q_-CASE bioluminescence resonance energy transfer (BRET) biosensor, and 1 μg/2 μL DNA/Lipofectamine 2000 ratio was used.^40^ The G_q_-CASE sensor reports receptor-mediated dissociation of Gα_q_βγ proteins through a decrease in BRET between a nanoluciferase-tagged Gα_q_ as donor and the cpVenus-tagged Gβγ serving as acceptor, enabling real-time monitoring of G_q_ signaling in live cells.

24 h after transfection, cells were harvested using trypsin and 300,000 cells/mL were plated in assay-compatible poly l-lysine-coated plates (CELL CULTURE MICROPLATE, 96 WELL, Greiner 655083). BRET assays were performed 48 h later. Prior to measurements, cells were washed with HBSS and incubated with the luciferase substrate Furimazine (10 μM, TargetMol Chemicals Inc., T15359) under assay buffer conditions (Opti-MEM, HEPES 10 mM) optimized to maintain receptor responsiveness. BRET signals were recorded using a VANTAstar plate reader (BMG Labtech, Germany). Baseline recordings were taken for 3 min and followed by stimulation with a TRAP peptide and recording for 60 min. Nluc and cpVenus173 emission intensities were measured using 450/40 nm and 535/30 nm monochromators, respectively. The integration time was set to 0.3 s in both channels. BRET ratios were defined as acceptor emission/donor emission. The basal BRET ratio before ligand stimulation (Ratio_basal_) was defined as the average of three consecutive BRET values. The ΔBRET was calculated for each well as a percent over basal ([(Ratio_stim_ – Ratio_basal_)/Ratio_basal_] × 100). Subsequently, the average ΔBRET of vehicle control was subtracted.

Data were collected from three independent experiments, each performed with three technical replicates. The ΔBRET ligand concentration–response curves were generated using GraphPad Prism 7.05 software fitted to a sigmoidal three-parameter logistic equation to quantify agonist potencies (pEC_50_), maximal responses (*E*_max_), and curves were constructed from the AUC of the kinetic traces normalized to the buffer-only (vehicle-treated) condition in each data set.

### PAR1 Surface Expression through Live-Cell Enzyme-Linked Immunosorbent Assay

To quantify cell surface receptor expression, PAR1 KO HEK293T cells were transfected with either empty pcDNA (control) or PAR1 WT/mutant constructs, with PAR1 KO cells serving as negative control. Following a 48 h growth period, the cells were washed once with 1% bovine serum albumin (BSA) in TBS. Cells were then incubated with a N-terminal binding mouse antihuman-PAR1 primary antibody (Santa Cruz, ATAP2: sc-13503; RRID: AB_2101175) in 1% BSA/TBS for 2 h at 4 °C. Following three washes with 1% BSA/TBS, cells were incubated for 2 h at 4 °C with a horseradish peroxidase (HRP)-conjugated horse antimouse secondary antibody (Cell signaling, 7076) diluted in 1% BSA/TBS. After three additional washes, 200 μL of 3,3′,5,5′-tetramethylbenzidine (TMB) substrate (Sigma-Aldrich, T8665) was added to each well. The reaction was allowed to proceed for 20 min before being quenched by the addition of 0.5 M of H_2_SO_4_. Absorbance was measured at 450 nm using a BMG VANTAstar plate reader.

## Supplementary Material



## Data Availability

All coordinate files, topology files, parameter files, and scripts necessary for running the MD simulations, and custom scripts for analyzing the MD data and plotting the analysis data are available at https://github.com/kuenzelab/PAR1_SwSoT.

## Notes

The Supporting Information includes tables defining the collective variables used in the SMwST simulations (Table S1), summarizing pEC_50_ and *E*_max_ values of PAR1 mutants (Table S2), summarizing the composition of the MD simulation systems (Table S3), and listing the primers used for site-directed mutagenesis of PAR1 (Table S4). Supporting figures include a structural comparison of the PAR1 AlphaFold model with reference GPCR structures (Figure S1), clustering of PAR1 and related GPCR structures based on the selected CVs (Figure S2), convergence analysis of the SMwST simulations (Figure S3), ligand rmsd box plots (Figure S4), PCA-based comparison of unbiased MD and SMwST ensembles (Figure S5), results of a PCA-based residue importance analysis (Figure S6), PAR1 activation-related structural descriptors along the string for the VPX + Na^+^ and tethered agonist-bound simulations (Figure S7), an MD snapshot together with a free-energy landscape illustrating chloride ion coordination at the KRK motif (Figure S8), and cell surface expression data of PAR1 WT and mutants (Figure S9) (PDF)
