# Supplementary material for: Energy Landscape Sampling Reveals Ligand-Dependent Structural Dynamics of the Protease-Activated Receptor 1
Source: J Chem Inf Model. 2026 Sep 23;66(19):12800–15. doi: 10.1021/acs.jcim.6c01315 (PMC13664097; doi:10.1021/acs.jcim.6c01315)
Supplement: Supplementary file 1 [file ci6c01315_si_001.pdf]

Supporting Information for

Energy Landscape Sampling Reveals Ligand-Dependent Structural Dynamics of the Protease-Activated Receptor 1

Inken Kaja Schwerin[1], Shihai Jiang[2], Claudia Stäubert[3], Berend Isermann[2,4,*], Georg Künze[1,5,6,*]

[1] Institute for Drug Discovery, Leipzig University, Brüderstraße 34, 04103 Leipzig, Germany

[2] Institute of Laboratory Medicine, Clinical Chemistry and Molecular Diagnostic, University Hospital Leipzig, Paul-List-Straße 13-15, 04103 Leipzig, Germany

[3] Rudolf Schönheimer Institute of Biochemistry, Molecular Biochemistry, Medical Faculty, Leipzig University, Johannisallee 30, 04103 Leipzig, Germany

[4] LeiCeM - Leipzig Center of Metabolism, Leipzig University, 04103 Leipzig, Germany

[5] Interdisciplinary Center for Bioinformatics, Leipzig University, 04107 Leipzig, Germany

[6] Center for Scalable Data Analytics and Artificial Intelligence, Leipzig University, 04105 Leipzig, Germany

* Correspondence to: georg.kuenze@uni-leipzig.de, berend.isermann@medizin.uni-leipzig.de

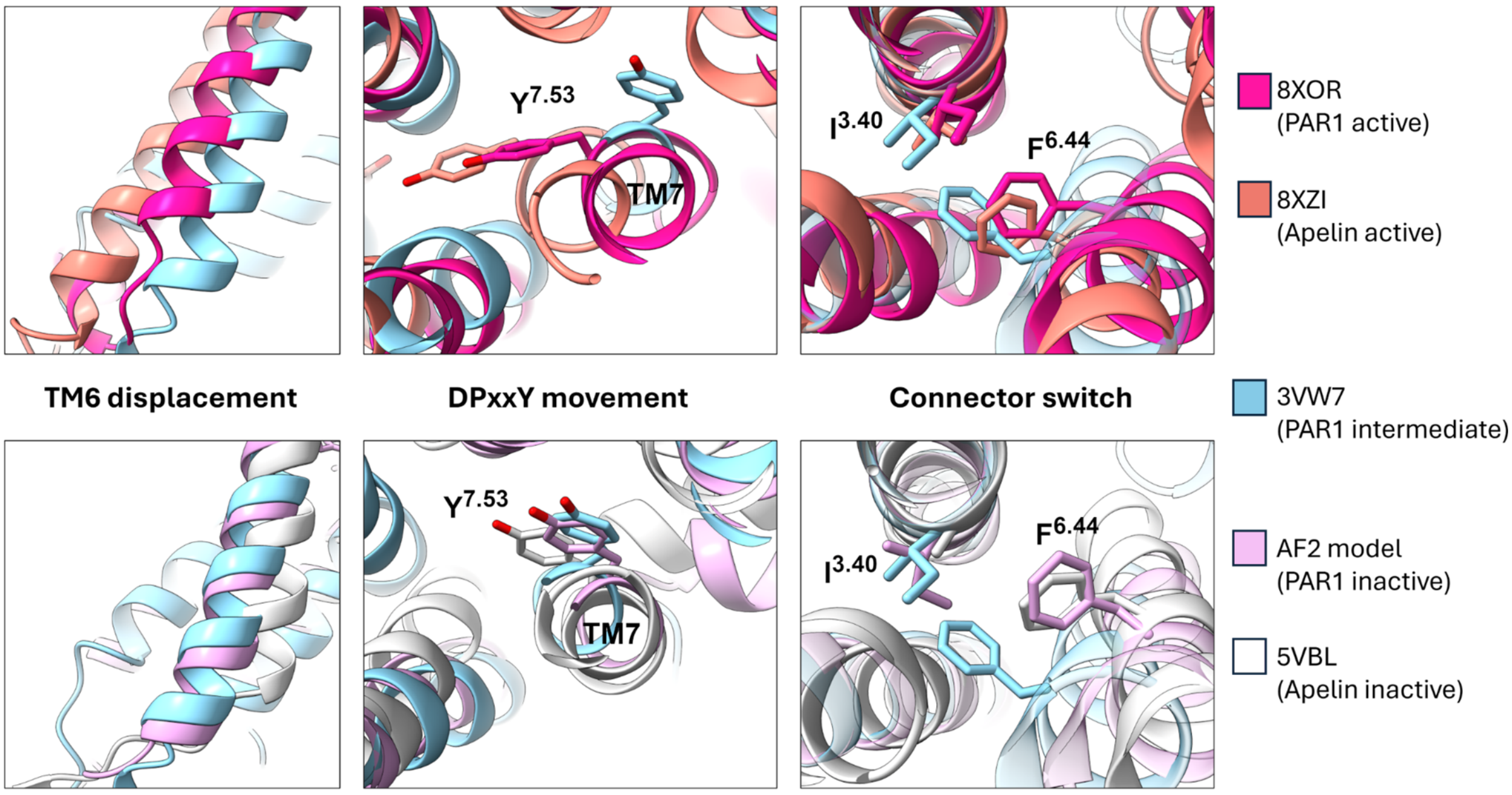

Figure S1: Validation of the AlphaFold2 PAR1 model as an inactive-state endpoint. The PAR1 active-state structure (PDB: 8XOR), VPX-bound intermediate-state structure (PDB: 3VW7), and the AlphaFold2 PAR1 model were compared with active and inactive reference structures of the related apelin receptor. Structural overlays are shown for TM6, the DPxxY motif, and the connector motif. The 3VW7 structure exhibits an intermediate-state character and contains an active-state-like connector motif, whereas the AlphaFold2 model adopts a more inactive-like conformation of the connector motif and other activation-related regions. ΔRMSD values, calculated as RMSD to the active-state reference minus RMSD to the inactive-state reference, were -0.083 Å for 3VW7 and 0.587 Å for the AlphaFold2 model, supporting the greater similarity of the AlphaFold2 model to inactive-state GPCR reference structures. Positive ΔRMSD values indicate greater similarity to the inactive-state reference structure, whereas negative values indicate greater similarity to the active-state reference structure.

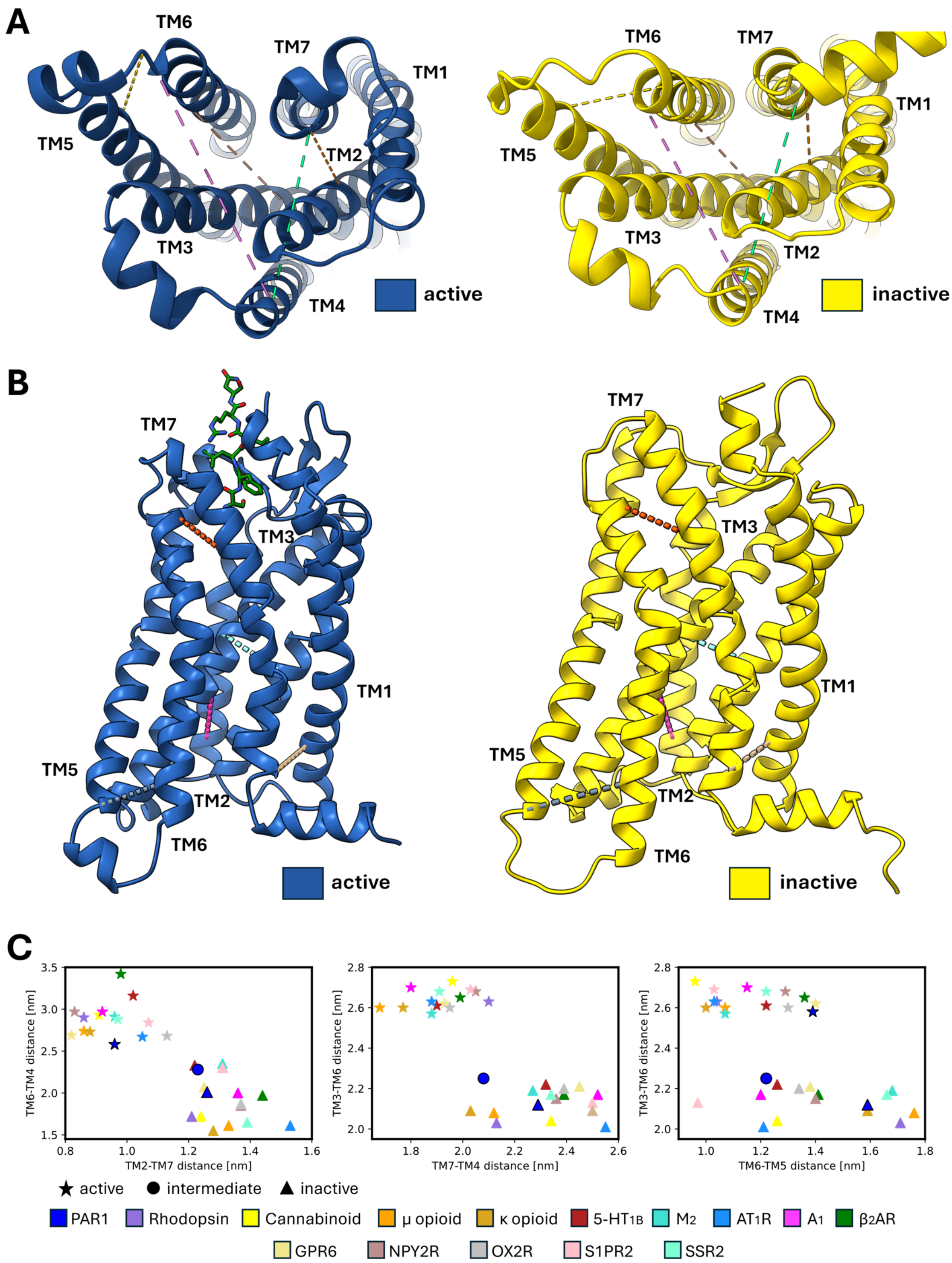

Figure S2: The five collective variables (CVs) employed in this study distinguish active and inactive conformations of PAR1 and other class A GPCRs. (**A**) Intracellular views of active (PDB: 8XOR) and inactive (AlphaFold2 model) PAR1 structures. Transmembrane helices are labeled, and the interhelical distances used as CVs are indicated by dashed lines. (**B**) Side views of the active- and inactive-state PAR1 structures showing the interhelical distances used to calculate the a100 activation index, indicated by dashed lines (see also the definition of the a100 index in Table S1). (**C**) Distribution of PAR1 and additional class A GPCR structures in CV space. Active- and inactive-state GPCR conformations, classified according to their GPCRdb activation-state annotation, are shown as stars and triangles, respectively. The CV values were either adapted from Fleetwood et al.[29] or calculated in this study from the corresponding GPCRdb-derived structures.

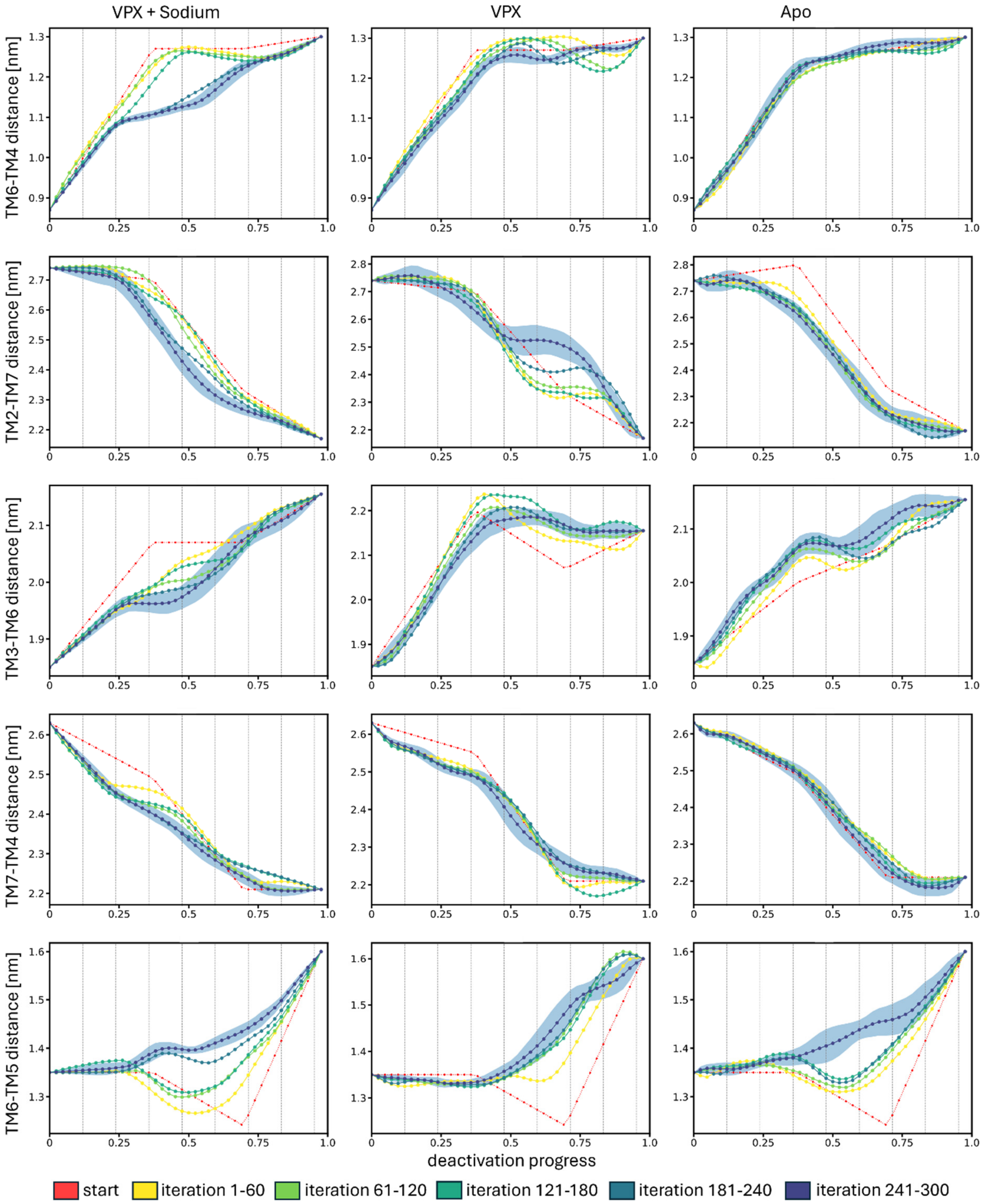

Figure S3: Convergence of strings across iterations of SMwST simulations for different PAR1 systems. Average strings from successive SMwST iterations are projected onto the original PAR1 CVs for the VPX + Na^+, VPX-bound, and apo receptor systems. The x-axis represents progression along the string from the active toward the inactive state. The blue shaded region denotes the standard deviation across the last 60 string iterations, illustrating convergence behavior.

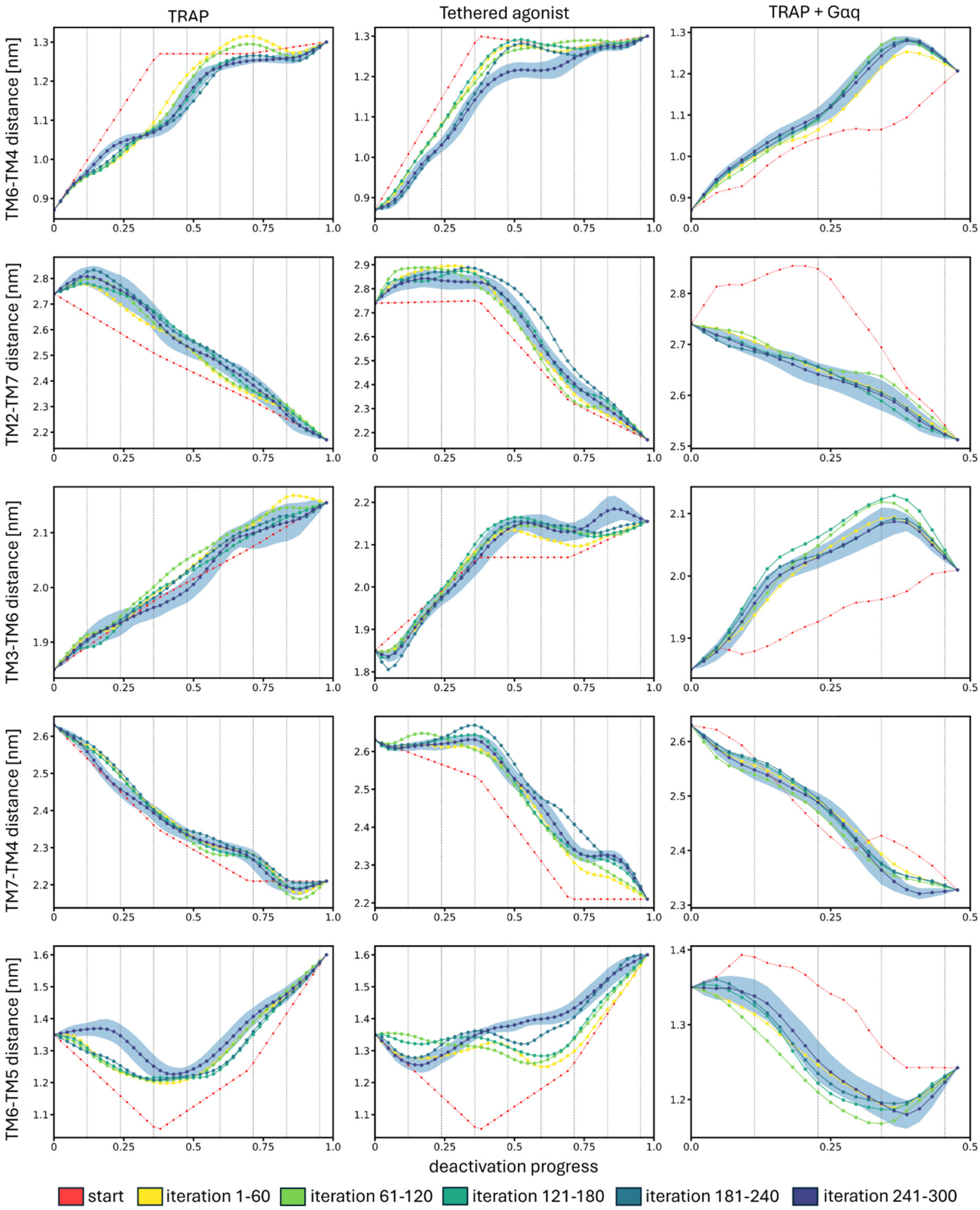

Figure S3 (continued): Convergence of strings across iterations of SMwST simulations for different PAR1 systems. Average strings from successive SMwST iterations are projected onto the original PAR1 CVs for the TRAP, TRAP+Gα_q, and tethered agonist systems. The x-axis represents progression along the string from the active toward the inactive state. The blue-shaded region denotes the standard deviation across the last 60 string iterations, illustrating convergence behavior.

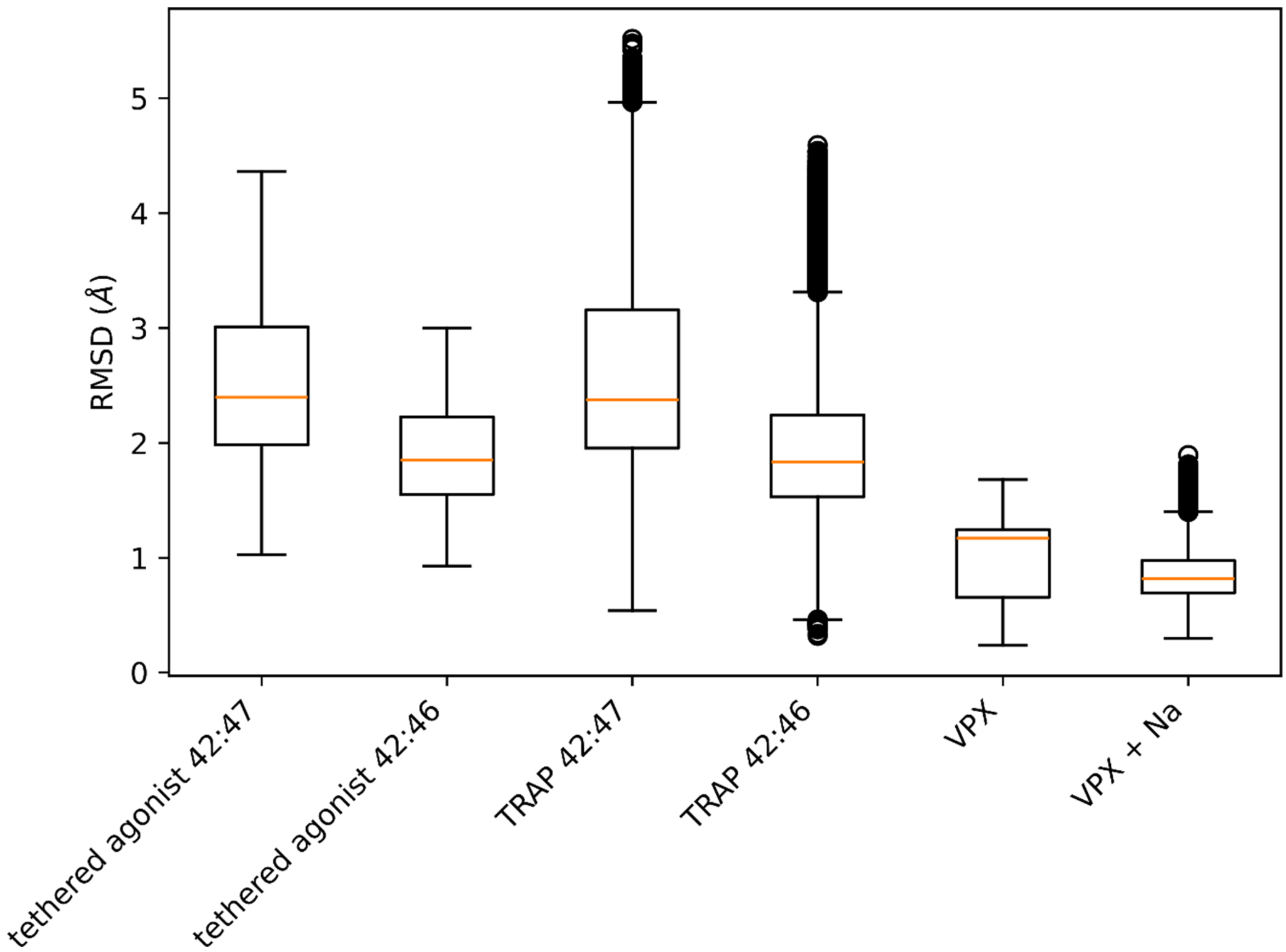

Figure S4: Ligand stability during SMwST simulations. Box plots showing ligand RMSD values for the tethered agonist, TRAP peptide, VPX, and VPX+Na^+ systems. RMSD values were calculated for all PAR1 conformations sampled along the final string during the last 100 iterations of the SMwST simulations relative to the respective starting structure. For the tethered agonist and TRAP peptide systems, RMSD values are shown both with and without inclusion of residue R47, which exhibits elevated flexibility and contributes to the overall ligand RMSD. The lower RMSD values obtained upon exclusion of R47 indicate that the remaining ligand residues remain stably bound throughout the simulations. In each box plot, the center line indicates the median, the box boundaries represent the interquartile range (IQR), and the whiskers extend to the most extreme data points within 1.5 × IQR. Points beyond the whiskers are shown as outliers.

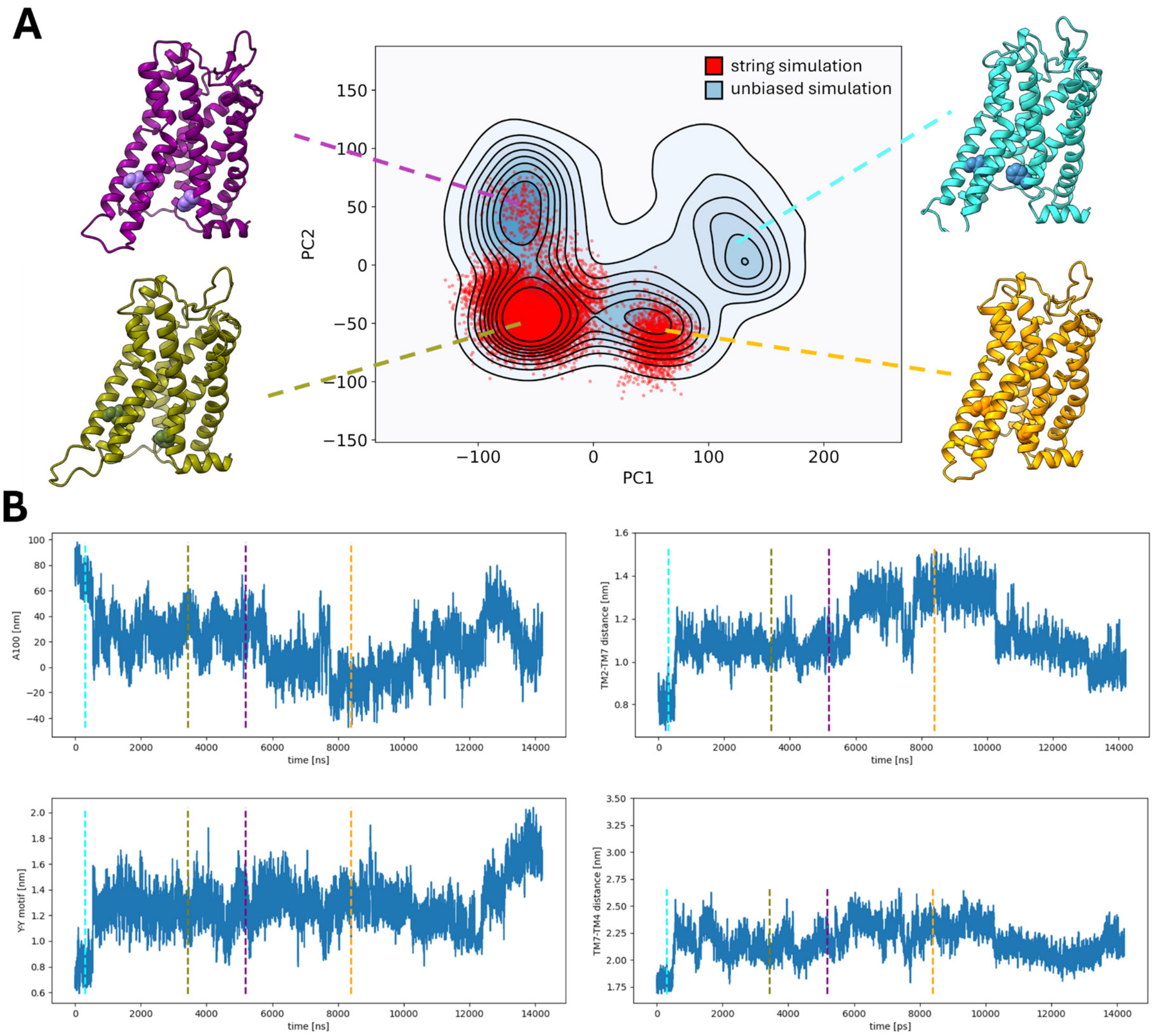

Figure S5. Comparison of conformational ensembles sampled by unbiased MD and SMwST simulations. (**A**) Projection of conformations sampled in the 15 μs unbiased MD simulation and in the SMwST simulations of the apo PAR1 receptor onto a common PC1–PC2 space. Representative PAR1 structures from four major regions of the PC space are shown next to the PCA plot. The cyan structure corresponds to an active-state conformation, the yellow structure to an inactive-state conformation, and the purple and olive-green structures to likely intermediate conformations. (**B**) Time evolution of four activation-related progress variables during the unbiased MD simulation: the a100 index, the Y–Y distance, the TM2–TM7 distance, and the TM4–TM7 distance. The dashed vertical lines indicate the time points corresponding to the representative structural snapshots shown in panel A.

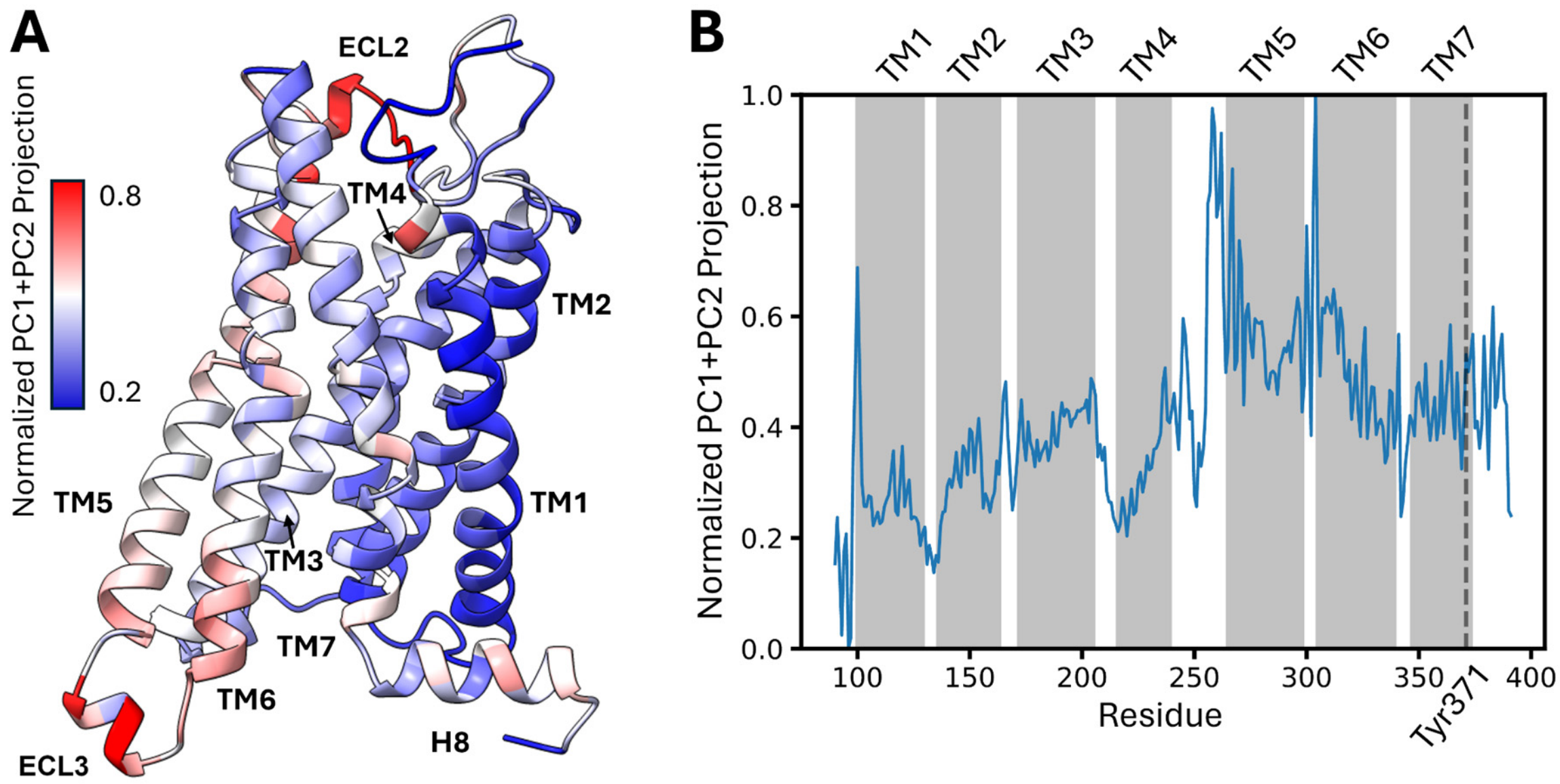

Figure S6: Principal component analysis of string simulations supports the importance of TM5, TM6, and TM7 movements in PAR1 activation. Back-projection of the principal component eigenvalues onto the inter-residue distance input features illustrates the relative contribution of each feature to the PCA. These importance values are mapped onto the PAR1 structure in (**A**) (red, high importance; blue, low importance) and plotted as a function of PAR1 residue number in (**B**).

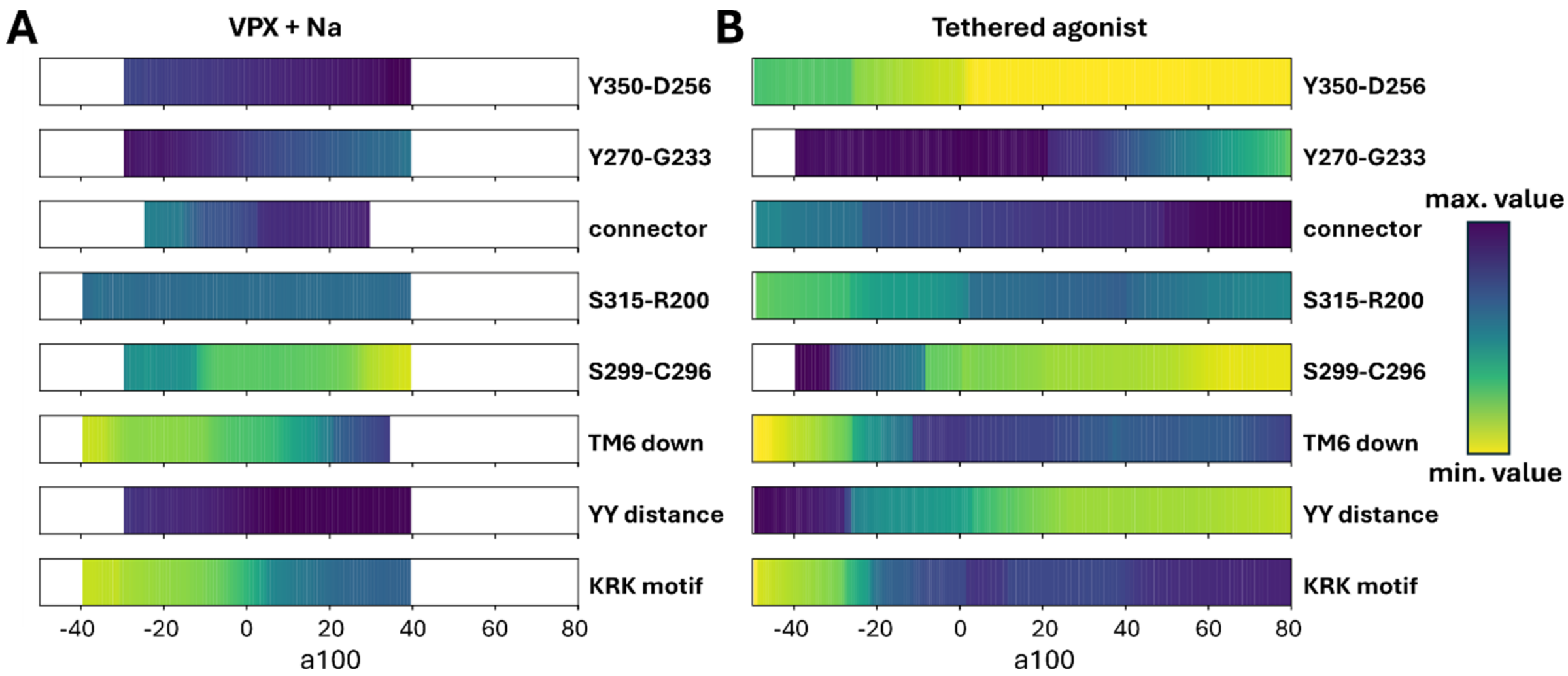

Figure S7: Ligand-dependent changes of structural descriptors associated with PAR1 activation in simulations with (A) VPX+Na^{+} and (B) the tethered agonist. Variations in the selected descriptors are represented as color bars, where dark blue indicates the highest observed values (distance or RMSD) and yellow indicates the lowest values sampled in the respective simulations. To relate these structural changes to the overall activation process, descriptor values are plotted as a function of the a100 activation index, which serves as the y-axis coordinate.

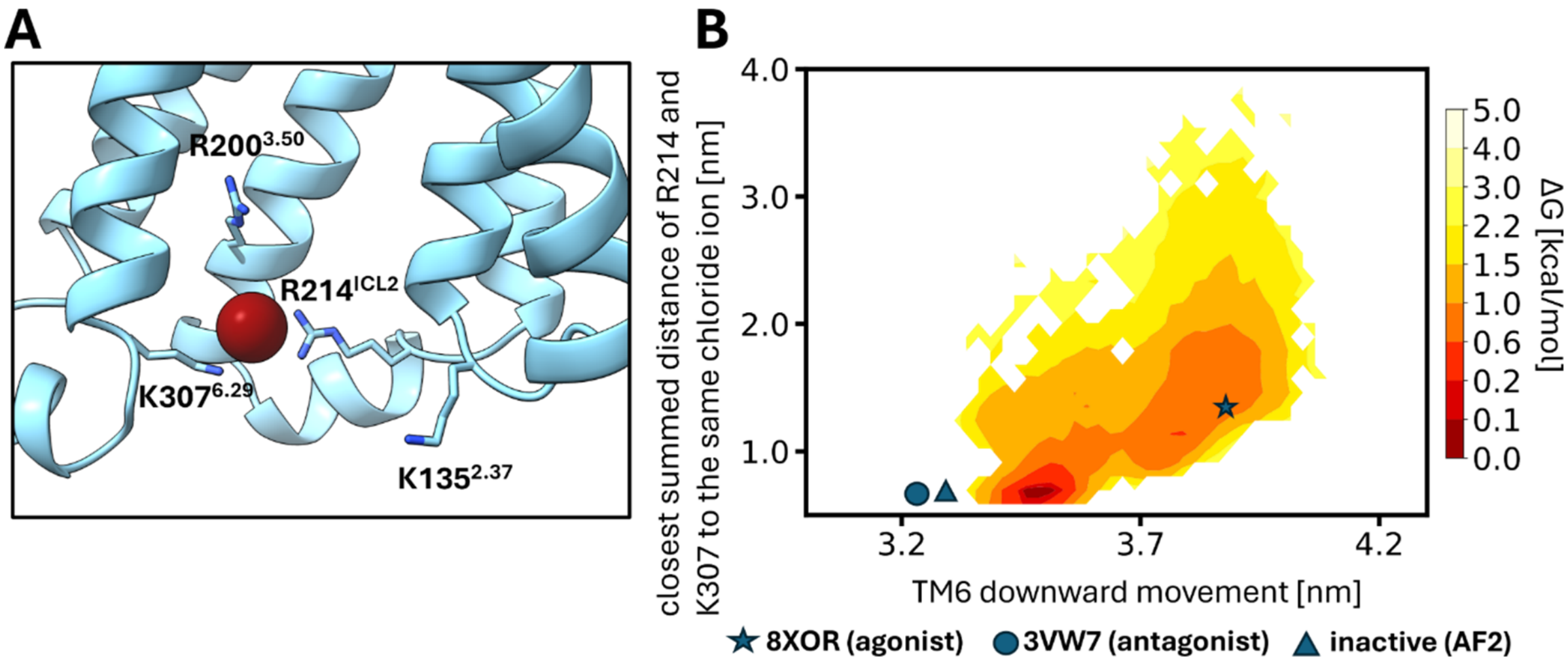

Figure S8: Chloride ion binding to the KRK motif is associated with inactive-state PAR1 conformations. (**A**) Representative MD snapshot illustrating coordination of a Cl^- ion by the KRK motif, with coordination completed by a fourth residue - R214. (**B**) Free energy landscape derived from the apo receptor simulation and projected onto descriptors of TM6 downward displacement and the minimum distance between the Cl^- ion and either R200 or K307.

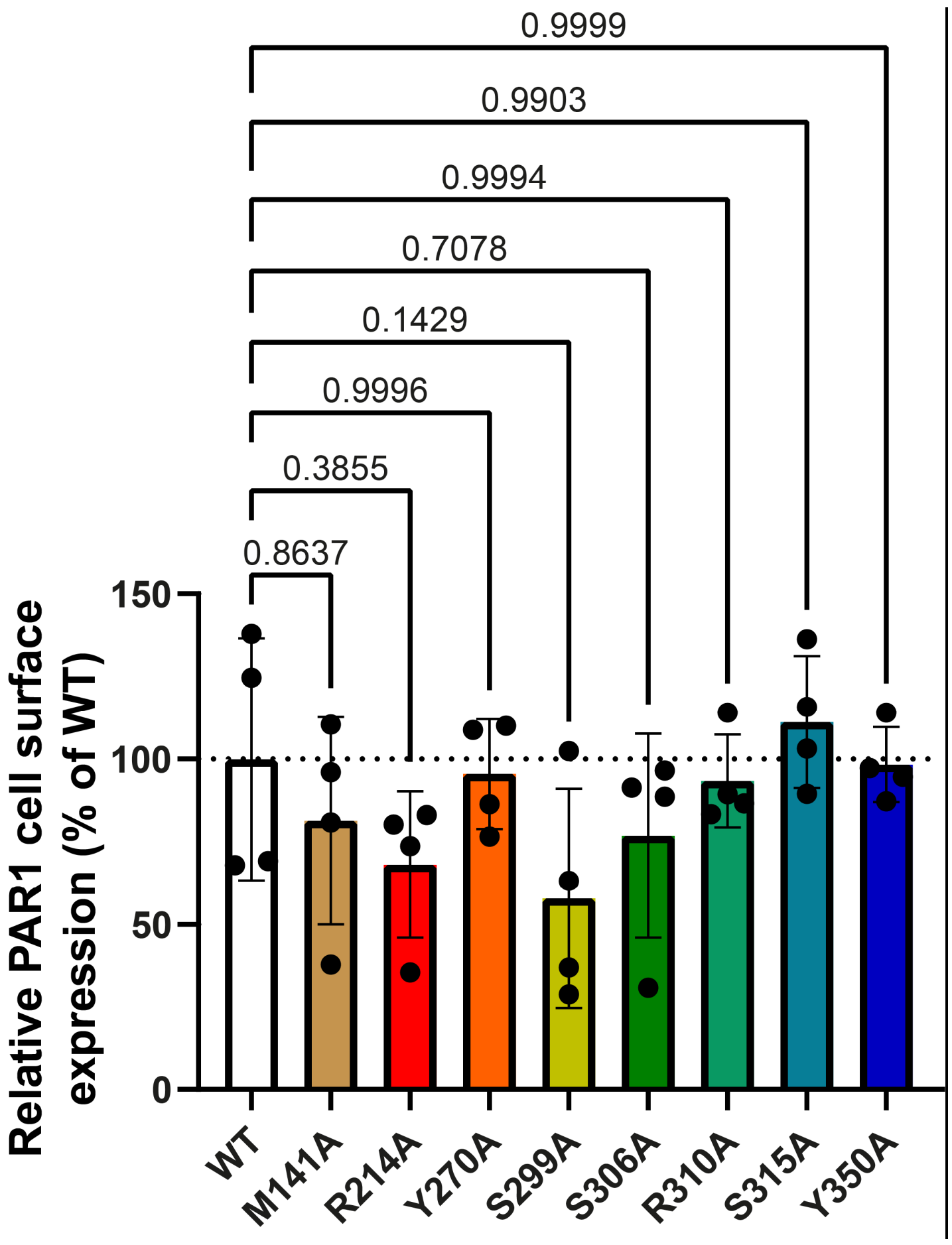

Figure S9: Cell surface expression of PAR1 WT and mutants in PAR1 KO HEK293T cells. OD values are normalized to WT PAR1 expression (ODraw = 2.0976 ± 0.6425) measured in a live-cell ELISA. Data represent mean ± SEM from n = 4 independent biological replicates. Statistical analyses were performed using a one-way ANOVA followed by a post-hoc Tukey's test. P-values relative to WT PAR1 are indicated above the corresponding bars.

Table S1: Definition of collective variables.

Measurement	Definition
TM2-TM7 distance	Cα-Cα distance between A145$^{2.47}$ and Y372$^{7.54}$
TM4-TM6 distance	Cα-Cα distance between L216$^{4.39}$ and K308$^{6.30}$
TM7-TM4 distance	Cα-Cα distance between Y372$^{7.54}$ and L216$^{4.39}$
TM3-TM6 distance	Cα-Cα distance between C184$^{3.34}$ and S315$^{6.37}$
TM6-TM5 distance	Cα-Cα distance between K307$^{6.29}$ and I294$^{5.62}$
a100 index	-14.43 * (A123$^{1.53}$ to Y373$^{7.55}$) - 7.62 (D148$^{2.50}$ to Y187$^{3.37}$) + 9.11 (L192$^{3.42}$ to A219$^{4.42}$) - 6.32 (S298$^{5.66}$ to L312$^{6.34}$) - 5.22 (H336$^{6.58}$ to Y353$^{7.35}$) (CA distances)
YY distance	distance Y290$^{5.58}$ CZ to Y371$^{7.53}$ CZ
D256-Y350	distance D256^{ECL2} OD to Y350$^{7.32}$ OH
Y270-G233	distance Y270$^{5.38}$ OH to G233$^{4.56}$ O
Connector	(I190$^{3.40}$, F322$^{6.44}$ RMSD to PDB 8XOR) – (I190$^{3.40}$, F322$^{6.44}$ RMSD to Inactive-State AlphaFold Model)
S315-R200	distance S315$^{6.37}$ OG to R200$^{3.50}$ NH2
C296-C299	distance C296$^{5.64}$ O to S299$^{5.67}$ OG
KRK motif	distance K307$^{6.29}$ NZ to K135$^{2.37}$ NZ
TM6 down	distance R305$^{6.27}$ CA to M186$^{3.36}$ CA
TM6 displacement	distance L312$^{6.34}$ CA to R200$^{3.50}$ CA

Table S2: pEC_{50} and E_{max} values of WT and mutant PAR1 receptors in TRAP6-induced G_q dissociation assays. The data are presented as means ± SEM from three independent experiments. Values in parentheses show 95 % CI. *P < 0.05, by one-way ANOVA followed by Fisher's LSD multiple comparisons test compared with WT PAR1.

	**pEC_{50}**	**E_{max}**	**Sample Size**
**WT**	7.567<br>(7.047 to 8.101)	-47.94<br>(-58.90 to -10.96)	3
**M141A**	9.196<br>(8.575 to 9.711)<br>*P<0.0001*	-23.20<br>(-21.80 to 2.025)<br>*P<0.0001*	3
**R214A**	5.686<br>(5.141 to 6.102)<br>*P=0.0001*	-30.22<br>(-34.95 to -4.732)<br>*P=0.0219*	3
**Y270A**	7.846<br>(6.827 to 10.13)<br>*P=0.1020*	-30.03<br>(-38.16 to -8.129)<br>*P=0.0296*	3

**S299A**	6.848<br>(6.404 to 7.358)<br>*P=0.4936*	-23.79<br>(-29.54 to -5.745)<br>*P<0.0001*	3
**S306A**	6.714<br>(6.297 to 7.132)<br>*P=0.1619*	-23.30<br>(-29.92 to -6.615)<br>*P<0.0001*	3
**R310A**	5.719<br>(4.789 to 6.346)<br>*P=0.0040*	-43.08<br>(-49.88 to -6.794)<br>*P=0.3499*	3
**S315A**	5.416<br>(3.897 to 5.854)<br>*P<0.0001*	-36.71<br>(-40.43 to -3.72)<br>*P=0.0462*	3
**Y350A**	6.803<br>(6.203 to 7.292)<br>*P=0.4067*	-23.61<br>(-28.33 to -4.718)<br>*P<0.0001*	3

Table S3: Overview of the MD system setup, including box size, number of atoms, replicate number, and total simulation time.

**Simulation**	**Box size (Å x Å x Å)**	**# Atoms for protein / POPC / waters / ions / small molecules**	**Replicates**	**Simulation time (μs)**
PAR1 Apo (unbiased)	83 x 83 x 103	4872 / 183 / 14640 / 74 / 0	1	14.2
PAR1 Apo (SMwST)	83 x 83 x 103	4872 / 183 / 14640 / 74 / 0	1	3.4
PAR1 + TRAP	83 x 83 x 105	4983 / 183 / 14640 / 76 / 0	1	3.4
PAR1 + Tethered ligand	97 x 97 x 123	5660 / 259 / 25900 / 129 / 0	1	3.4
PAR1 + VPX	83 x 83 x 103	4857 / 180 / 14400 / 73 / 69 (VPX)	1	3.4
PAR1 + VPX + Na	83 x 83 x 103	4857 / 181 / 14480 / 75 / 69 (VPX)	1	3.4
PAR1 + TRAP + Gα_q	103 x 103 x 163	10736 / 271 / 38016 / 208 / 0	1	1.7

Table S4: Site-directed mutagenesis primers used in this study

Bases	Primer name	Squence
M141A	M141A:1_FWD	GGTGGTGTACgcgCTGCACCTGG
	M141A:1_REV	GCCGGCTTCTTGACC
R214A	R214A:1_FWD	CCTCTCCTGGgcgACTCTGGGAAG
	R214A:1_REV	GACTGCATGGGATAC
Y270A	Y270A:1_FWD	CTATGCCTACgcgTTCTCAGCCTTCTCTG
	Y270A:1_REV	TAGCCTTCGAGCAGG
S299A	S299A:1_FWD	ATGTCTTAGCgcgTCCGCAGTTG
	S299A:1_REV	CGAATGATAGACACATAACAG
S306A	S306A:1_FWD	TGCCAACCGCgcgAAGAAGTCCC
	S306A:1_REV	ACTGCGGAAGAGCTAAG
R310A	R310A:1_FWD	CAAGAAGTCCgcgGCTTTGTTCCTGTC
	R310A:1_REV	CTGCGGTTGGCAACTG
S315A	S315A:1_FWD	TTTGTTCCTGgcgGCTGCTGTTT
	S315A:1_REV	GCCCGGGACTTCTTG
Y350A	Y350A:1_FWD	AGAGGCTGCCgcgTTTGCCTACCTC
	Y350A:1_REV	GTGGTGGAAGTGTGAG
